# Real-world effectiveness and safety of ranibizumab for the treatment of myopic choroidal neovascularization: Results from the LUMINOUS study

**DOI:** 10.1371/journal.pone.0227557

**Published:** 2020-01-21

**Authors:** Robin D. Hamilton, Andreas Clemens, Angelo Maria Minnella, Timothy Y. Y. Lai, Hong Dai, Taiji Sakamoto, Chui Ming Gemmy Cheung, Nor Fariza Ngah, Cornelia Dunger-Baldauf, Frank G. Holz, on behalf of the LUMINOUS study group

**Affiliations:** 1 Department of Medical Retina, Moorfields Eye Hospital NHS Foundation Trust and National Institute for Health Research (NIHR) Biomedical Research Centre (BRC) at Moorfields Eye Hospital, London, United Kingdom; 2 Medical Affairs Region Europe, Ophthalmology, Novartis Pharma AG, Basel, Switzerland; 3 Department of Cardiology and Angiology I, Heart Center Freiburg University, Faculty of Medicine, University of Freiburg, Freiburg, Germany; 4 Department of Ophthalmology, Catholic University of Sacred Hearth—Foundation “Policlinico Universitario A. Gemelli"—IRCCS, Rome, Italy; 5 Department of Ophthalmology & Visual Sciences, The Chinese University of Hong Kong, Hong Kong Eye Hospital, Hong Kong SAR, China; 6 Department of Ophthalmology, Beijing Hospital, National Center of Gerontology, Beijing, China; 7 Department of Ophthalmology, Kagoshima University Graduate School of Medical and Dental Sciences, Kagoshima, Japan; 8 Singapore Eye Research Institute, Singapore National Eye Centre, Singapore; 9 Hospital Shah Alam, Selangor, Malaysia; 10 Department of Ophthalmology, University of Bonn, Bonn, Germany; University of Florida, UNITED STATES

## Abstract

**Purpose:**

To assess the 1-year effectiveness, safety, and treatment patterns of ranibizumab in patients with myopic choroidal neovascularization (mCNV) enrolled in the LUMINOUS study.

**Methods:**

This 5-year, prospective, multicenter, observational, study enrolled 30,138 patients across all approved ranibizumab indications from outpatient ophthalmology clinics. 297 consenting patients (≥18 years) with mCNV who were treatment-naïve or prior-treated with ranibizumab or other ocular treatments were enrolled, and treated with ranibizumab according to the local product label. The main outcomes are visual acuity (VA; Early Treatment Diabetic Retinopathy Study letters or equivalent), adverse events during the study, and treatment exposure over 1 year. Results are presented by prior treatment status of the study eye and injection frequency.

**Results:**

Of the 297 mCNV patients recruited in the study, 108 were treatment-naïve and 175 were prior ranibizumab-treated. At baseline, the mean age of patients was 57.6 years, and 59.0 years and 80.6% and 65.7% were female in the treatment-naïve and prior ranibizumab-treated groups, respectively. Most were Caucasian (treatment-naïve, 88.9%; prior ranibizumab-treated, 86.9%). The mean (±standard deviation [SD]) VA letter changes to 1 year were +9.7 (±17.99) from 49.5 (±20.51) and +1.5 (±13.15) from 58.5 (±19.79) and these were achieved with a mean (SD) of 3.0 (±1.58) and 2.6 (±2.33) injections in the treatment-naïve and prior ranibizumab-treated groups, respectively. Presented by injection frequencies 1–2, 3–4 and ≥5 injections in Year 1, the mean (SD) VA changes were +15.0 (±14.70), +7.7 (±19.91) and −0.7 (±16.05) in treatment-naïve patients and +1.5 (±14.57), +3.1 (±11.53) and −3.6 (±11.97) in prior ranibizumab-treated patients, respectively. The safety profile was comparable with previous ranibizumab studies.

**Conclusions:**

Ranibizumab treatment for mCNV showed robust VA gains in treatment-naïve patients and VA maintenance in prior ranibizumab-treated patients in a clinical practice setting, consisting mainly of Caucasians. No new safety signals were observed during the study.

## Introduction

Pathologic myopia is characterized by the elongation of axial length and degenerative changes in the fundus associated with visual loss [1, 2]. Myopic CNV (mCNV), is the most common, bilateral sight-threatening complication of pathologic myopia, [3] and presents with macular hemorrhage, serous retinal detachment and subretinal hemorrhage with fibrotic membrane formation [4]. It is common in the working-age population (aged less than 50 years), more prevalent in Asians than Caucasians; [3, 5–7] it can lead to irreversible vision loss if left untreated [8–14].

Anti-vascular endothelial growth factor (anti-VEGF) agents are the first choice of treatment for mCNV [15–17]. Ranibizumab 0.5 mg was the first anti-VEGF agent approved for the treatment of visual impairment due to CNV secondary to pathologic myopia in 2013 in the European Union and in 2017 in the United States, based on the results of the RADIANCE (**R**anibizumab **A**nd P**D**T [verteporf**I**n] ev**A**luation i**N** myopic **C**horoidal n**E**ovascularization) trial [18–21]. Aflibercept was also approved for the treatment of mCNV [22]. However, the results of randomized controlled trials (RCTs) are not always reflective of the real-world performance of a therapy mainly due to strict eligibility criteria, regular frequent follow-ups, and different treatment intensity observed. Thus, the LUMINOUS™ (NCT01318941) study was designed to evaluate the effectiveness and safety of ranibizumab in broader patient populations in real-world clinical practice across all the approved indications. The 1-year global and country-specific efficacy, safety outcomes and treatment patterns observed in the treatment-naïve and prior ranibizumab-treated patients with mCNV enrolled in LUMINOUS are presented here.

## Methods

### Study design

LUMINOUS was a 5-year (March 2011 to April 2016), prospective, observational, multicenter, open-label, single-arm, global study, conducted at 488 clinical sites across 42 countries. Though enrollment commenced from March 2011, mCNV patients were enrolled only from July 2013 since ranibizumab was approved for the treatment of patients with visual impairment due to CNV secondary to pathologic myopia in the European Union in May 2013 [23].

The study was conducted in accordance with the Guidelines for Good Pharmacoepidemiology Practices issued by the International Society for Pharmacoepidemiology, with any applicable national guidelines, and ethical principles laid down in the Declaration of Helsinki. The study protocol was reviewed and approved by an Independent Ethics Committee or Institutional Review Board (**S1 Table**) for each center. All participants provided written informed consent.

### Participants

Consenting adult (aged ≥18 years) patients, who were either treatment-naïve, or prior-treated with ranibizumab or another ocular therapy for any of the approved indications included in the local product label, were enrolled. Patients were excluded if they were participating in other investigational studies or if they had received systemic anti-VEGF therapy other than ranibizumab 90 days or ocular anti-VEGF therapy 30 days prior to enrollment. Further details of the inclusion and exclusion criteria are provided in **S1 Methods**.

### Treatments

Patients were treated with intravitreal ranibizumab 0.5 mg according to the local product label. Further retreatments were performed at clinicians’ discretion. The first eye treated during the study was considered the primary treated eye. If both eyes were first treated on the same date, or if both eyes were pre-treated, the eye with the earliest diagnosis date was considered the primary treated eye. Further details are provided in **S1 Methods.**

### Study objectives and endpoints

The primary objective of LUMINOUS was to describe the safety and effectiveness of ranibizumab in real world clinical practice. Secondary objectives included the assessment of treatment patterns of ranibizumab in routine clinical practice and quality of life. The primary effectiveness variable was the mean change in visual acuity (VA) (preferably best-corrected VA [BCVA]) from baseline for the primary treated eye set. The number of ranibizumab injections administered overall, over 1 year, the treatment patterns, and safety over 1 year were reported. Optical coherence tomography (OCT) data were not available for all patients, varied in its nature and instrumentation at different sites and hence OCT parameters were not analyzed as end points.

### Assessments and analysis

The demographic data and primary indication for initiation of ranibizumab treatment were collected from the patients at baseline. These data were presented by pre-treatment status, indication, and time period. Further details are provided in **S1 Methods.**

### Statistical analysis

All effectiveness and safety data were summarized descriptively. For treatment-naïve eyes, the date of first on-study injection with ranibizumab was considered the baseline date (Day 1). The baseline date for the primary treated eye was the date of study entry if the primary treated eye had been pre-treated with ranibizumab. Effectiveness data were presented only for patients in the primary treated eye set for whom baseline and 1-year data were captured (365 days after the baseline date [±45 days; Day 319 to Day 409]). The mean change in VA from baseline at 1 year was analyzed by baseline injection frequencies (1–2, 3–4, and ≥5) and baseline VA category (<23, 23–<39, and 39–<60, 60–<74, and ≥74 letters). The number of injections and monitoring visits up to 1 year were summarized for patients who had participated in the study for at least 365 days.

Safety was assessed based on the incidence rate, relationship, and severity of treatment-emergent ocular and non-ocular adverse events (AEs). Ocular (for the primary treated eye) and non-ocular AEs presented by pre-treatment for the entire study period.

## Results

### Patient disposition and baseline characteristics

The LUMINOUS study enrolled 30,138 patients across all approved indications (neovascular age-related macular degeneration [nAMD], diabetic macular edema [DME], branch retinal vein occlusion [BRVO], central RVO [CRVO] and mCNV) worldwide. In this analysis, 297 patients with mCNV were enrolled who were either treatment-naïve (n = 108) or prior ranibizumab-treated (n = 175). There were 14 patients who were treated with ocular treatments other than ranibizumab before entering LUMINOUS. Overall, 80 (26.9%) patients discontinued the study. The main reasons for study discontinuation were loss to follow-up (10.4%, [n = 31]), patients no longer required study drug (5.1%, [n = 15]), and protocol deviation (4.4%, [n = 13]). Patients might have continued in the study but were not included in the analysis if they had no visits in a window around 365 days after the baseline date [±45 days; Day 319 to Day 409]. The primary treated eye set included 59 patients in the treatment-naïve and 119 patients in the prior ranibizumab-treated groups who had both baseline and Month 12 VA. The safety set comprised of 108 treatment-naïve and 175 prior ranibizumab-treated patients who were treated with ≥1 dose of ranibizumab during this study or prior to the start of study and had ≥1 safety assessment after the first treatment.

At baseline, the mean (±standard deviation [SD]) age was 57.6 (±15.6) years in the treatment-naïve and 59.0 (±14.9) years in the prior ranibizumab-treated patients. In both groups, the majority were female (treatment-naïve, 80.6% and prior ranibizumab-treated, 65.7%) and Caucasian (treatment-naïve, 88.9% and prior ranibizumab-treated, 86.9%). The patient demographics and baseline characteristics (baseline VA: 49.5 [±20.51] letters in treatment-naïve patients and 58.5 [±19.79] in prior ranibizumab-treated group) are summarized in **Table 1.** The countries that enrolled the most number of treatment-naïve patients with mCNV were Poland (n = 12), Russia (n = 12), United Kingdom (UK; n = 11), and Spain (n = 7). The median time from diagnosis to treatment was 14.5 days in the treatment-naïve patients and 444 days in the prior ranibizumab-treated patients.

**Table 1:** Baseline demographics, disease, and ocular characteristics for treatment-naïve and prior ranibizumab-treated patients with mCNV.

Characteristics	Treatment-naïve, n = 108	Prior ranibizumab-treated, n = 175
**Patient demographics**		
Mean (SD) age, years	57.6 (15.6)	59.0 (14.9)
Gender, %		
Male	19.4	34.3
Female	80.6	65.7
Race, %		
Caucasian	88.9	86.9
Black	0.9	0
Asian	4.6	9.1
Native American	1.9	0
Other	1.9	4.0
Missing	1.9	0
**Ocular characteristics**		
VA		
n	59	119
Mean (SD) VA, ETDRS letters	49.5 (20.51)	58.5 (19.79)
Median time from diagnosis to first treatment (treatment-naïve), to study entry (prior ranibizumab-treated), days	14.5	444

ETDRS, Early treatment diabetic retinopathy study; mCNV, myopic choroidal neovascularization; n, number of patients; SD, standard deviation; VA, visual acuity.

### Efficacy

**Fig 1:**
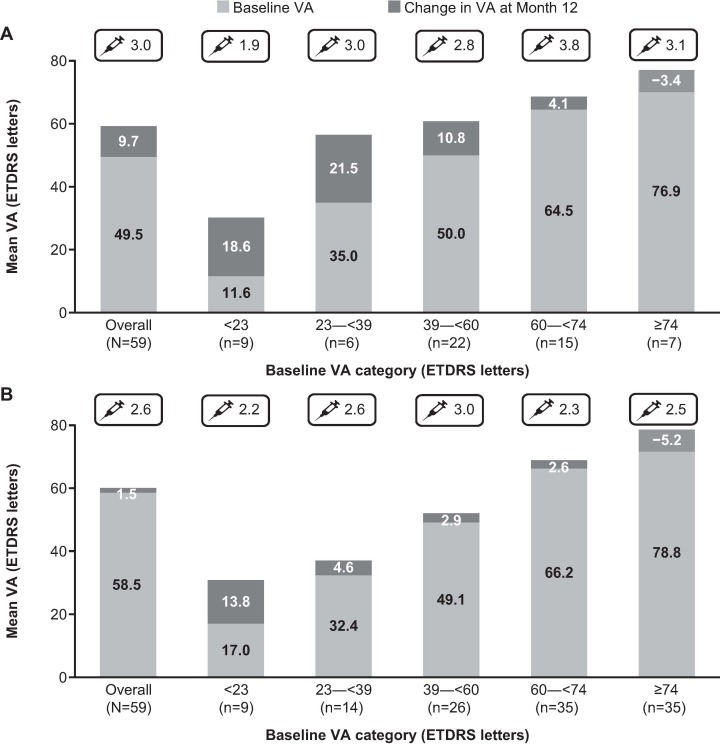
Mean change in VA at Month 12 based on baseline VA categories. **A) in treatment-naïve patients with mCNV. B) in prior ranibizumab-treated patients with mCNV.** Observed data set for VA change (primary treated eye set). n = number of evaluable patients with baseline and Month 12 data. The mean number of injections from baseline to Month 12 is designated with a syringe ETDRS, Early treatment diabetic retinopathy study; mCNV, myopic choroidal neovascularization; n, number of patients; VA, visual acuity.

The mean (SD) VA letter changes to 1 year were +9.7 (±17.99) from 49.5 (±20.51) in treatment-naïve patients and +1.5 (±13.15) from 58.5 (±19.79) in prior ranibizumab-treated patients, respectively. The mean (SD) VA of treatment-naïve patients (59.1 [±21.05]) and prior ranibizumab-treated patients (59.9 [±19.58]) was similar at Month 12. (**Fig 1A and 1B**). When stratified by baseline VA <23, 23–<39, and 39–<60, 60–<74 and ≥74 letters, mean VA changes after 1 year of ranibizumab treatment were +18.6, +21.5, +10.8, +4.1, and −3.4 letters in the treatment-naïve patients and +13.8, +4.6, +2.9, +2.6, and −5.2 letters in the prior ranibizumab-treated patients, respectively **(Fig 1A and 1B).**

**Fig 2:**
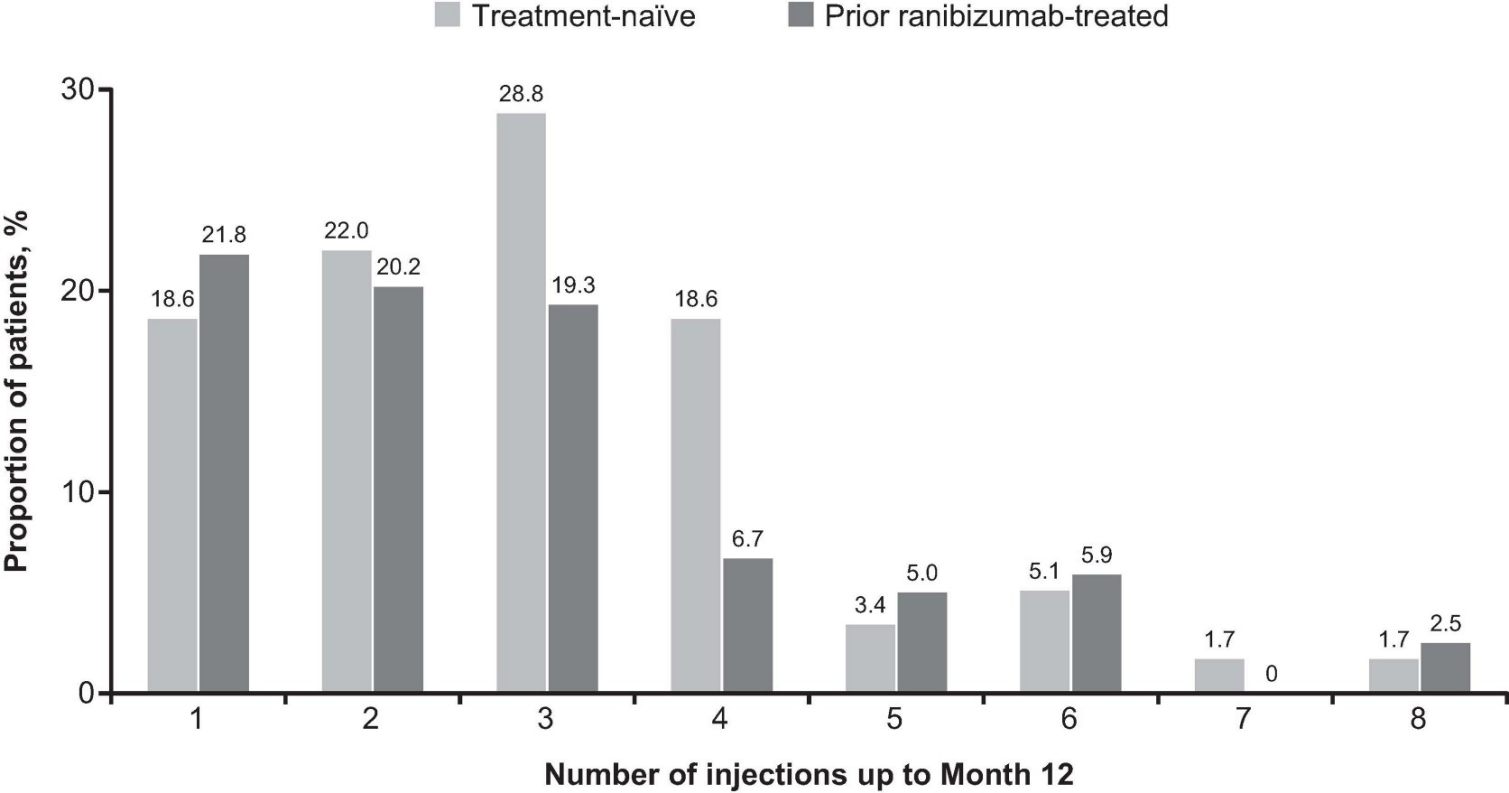
Frequency of injections over 12 months in treatment-naïve and prior ranibizumab-treated patients with mCNV. Observed data set for VA change (primary treated eye set). n = number of evaluable patients with baseline and Month 12 data. mCNV, myopic choroidal neovascularization; n, number of patients; VA, visual acuity.

When presented by injection frequencies over 1 year, the mean change in VA letter score was higher in the treatment-naïve patients who received 1–2 injections (+15.0, n = 24) compared with patients who received 3–4 (+7.7, n = 28) and ≥5 injections (−0.7, n = 7). The mean change in VA letter score was lower in the prior ranibizumab-treated group, with changes of +1.5 (n = 50), +3.1 (n = 31), and −3.6 (n = 19) in patients who received 1–2, 3–4, and ≥5 injections, respectively. During the first year of treatment, 69.5% of treatment-naïve and 77.3% of prior ranibizumab-treated patients received ≤3 injections (**Fig 2**).

### Treatment exposure and visits

The mean (SD) number of ranibizumab injections and monitoring visits were comparable in both the treatment-naïve (3.0 [±1.6] and 7.3 [±2.3], respectively) and prior ranibizumab-treated groups (2.6 [±2.3] and 7.6 [±3.2], respectively). When stratified by baseline VA <23, 23–<39, and 39–<60, 60–<74, and ≥74 letters, the mean number of injections in the treatment-naïve group was lowest in the <23 letters category (1.9) and was comparable between the other baseline VA categories (range 2.8–3.8). In the prior ranibizumab-treated group, the mean number of injections ranged from 2.2–3.0 and was comparable between the categories.

### Country-specific analysis of visual acuity outcomes and treatment patterns

The mean VA gain at 1 year and the mean number of ranibizumab injections varied across the countries that enrolled the highest number of treatment-naïve patients with mCNV (**S1 Fig**). In the treatment-naïve group, the mean change in VA letter score was highest in Poland (+20.1, from a baseline of 49.5, n = 12), followed by Spain (+12.3, from a baseline of 51.4, n = 7) with a mean (SD) of 2.8 (±1.14) and 2.1 (±1.77) injections, respectively. By contrast, the mean VA letter score changes in UK (n = 11) and Russia (n = 12) were +6.5 (baseline VA = 57.8) and +3.1 (baseline VA = 46.8), with a mean (SD) of 4.5 (±1.86) and 2.0 (±1.04) injections, respectively. When presented by injection frequencies, the mean change in VA letter score in the 1–3 injections category was +24.1 (n = 9), +15.2 (n = 7), + 16.3 (n = 4), and +4.2 (n = 11) in Poland, Spain, UK, and Russia, respectively. The mean change in VA letter score in the ≥4 injections category was +8.0 (n = 3) in Poland, +0.9 (n = 7) in UK, −9 (n = 1) in Russia, and −5 (n = 1) in Spain. The median time from diagnosis to treatment was 4 days each in Poland and Spain, 12 days in the UK, and 38 days in Russia (**S1 Fig**).

### Safety

Overall, in treatment-naïve patients with mCNV, ocular AEs were reported in 10.19% (n = 11) of patients, with increased intraocular pressure (IOP; 1.9%; n = 2) being the most common. In the prior ranibizumab-treated group ocular AEs were reported in 10.29% (n = 18) of patients, with the most common being glaucoma (1.71% [n = 3]), increased IOP, cataract, reduced VA, and conjunctival hemorrhage (each 1.14% [n = 2]) (**Table 2**). The incidence of ocular serious AEs (SAEs) was 0.93% in the treatment-naïve group. In the prior ranibizumab-treated group, the incidence of ocular SAEs was 1.14% (n = 2); retinal detachment and subretinal fibrosis were reported in one patient each (0.57%) (**Table 3**).

**Table 2:** Proportion of patients with ocular and non-ocular AEs in treatment-naïve and prior ranibizumab-treated patients with mCNV at 1 year.

Preferred term, n (%)	Treatment-naïve, n = 108	Prior ranibizumab-treated, n = 175
**Ocular AEs, total**	**11 (10.19)**	**18 (10.29)**
Increased IOP	2 (1.85)	2 (1.14)
Cataract	1 (0.93)	2 (1.14)
Glaucoma	0	3 (1.71)
Visual acuity reduced	1 (0.93)	2 (1.14)
Conjunctival hemorrhage	0	2 (1.14)
**Non-ocular AEs, total**	**11 (10.19)**	**23 (13.14)**
Depression	0	4 (2.29)
Nasopharyngitis	0	2 (1.14)
Fall	0	2 (1.14)
Localized infection	0	2 (1.14)
Pulmonary fibrosis	0	2 (1.14)

Indication and pre-treatment status refers to the primary treated eye. Only AEs occurring during the safety observation period are included. Preferred terms are presented by descending order of frequency in the total column. A patient with multiple occurrences of an AE was counted once per preferred term. A patient with multiple AEs is counted only once in the total row. Patients with a baseline visit date present are included. Data collected until the last recorded follow-up date was used to perform the analyses. Ocular and non-ocular AEs >2 in number are shown.

AE, adverse events; IOP, intraocular pressure; mCNV, myopic choroidal neovascularization; n, number of patients; VA, visual acuity.

**Table 3:** Proportion of patients with ocular and non-ocular SAEs in treatment-naïve and prior ranibizumab-treated patients with mCNV at 1 year.

Preferred term, n (%)	Treatment-naïve, n = 108	Prior ranibizumab-treated, n = 175
**Ocular SAEs, total**	1 (0.93)	2 (1.14)
Retinal detachment	0	1 (0.57)
Retinal pigment epithelial tear	1 (0.93)	0
Subretinal fibrosis	0	1 (0.57)
**Non-ocular SAEs, Total**	**3 (2.78)**	**3 (1.71)**
Cellulitis	1 (0.93)	0
Cerebrovascular accident	1 (0.93)	0
Death	0	1 (0.57)
Emphysema	0	1 (0.57)
Heart valve incompetence	0	1 (0.57)
Inappropriate antidiuretic hormone secretion	0	1 (0.57)
Myocardial ischemia	1 (0.93)	0
Pulmonary fibrosis	0	1 (0.57)

Indication and pre-treatment status refers to the primary treated eye. Only SAEs occurring during the safety observation period are included. Preferred terms are presented by descending order of frequency in the total column. A patient with multiple occurrences of a SAE was counted once per preferred term. A patient with multiple SAEs is counted only once in the total row. Patients with a baseline visit date present are included. Data collected until the last recorded follow-up date was used to perform the analyses.

mCNV, myopic choroidal neovascularization; n, number of patients; SAE, serious adverse events.

Non-ocular AEs were reported in 10.19% of treatment-naïve patients. One patient (0.93%) in this group reported myalgia, which was suspected to be related to ranibizumab.

Among the prior ranibizumab-treated patients, non-ocular AEs were reported in 13.14%. Depression (2.29%, n = 4), fall, nasopharyngitis, pulmonary fibrosis, and localized infection (each 1.14%, n = 2) were the most commonly reported non-ocular AEs (**Table 2**). Non-ocular SAEs were observed in 2.78% (n = 3) of the treatment-naïve group; cellulitis, cerebrovascular accident, and myocardial ischemia were reported in one patient each (0.93%). No cases of endophthalmitis were reported. The incidence of non-ocular SAEs in the prior-treated group was 1.71% (n = 3); emphysema, pulmonary fibrosis, heart valve incompetence, inappropriate antidiuretic hormone secretion, and death were reported in one patient each (**Table 3**).

There was one death of unknown cause that led to the discontinuation of ranibizumab but this was reported not to be related to ranibizumab. No patients in this study had a non-ocular SAE suspected of being related to ocular injection and/or ranibizumab. There were no ocular SAEs that led to the discontinuation of ranibizumab in the study.

The ocular AEs suspected to be related to ranibizumab were reported in 2.78% (n = 3) of treatment-naïve patients (**S2 Table**). In the prior ranibizumab-treated group, the ocular AEs suspected to be related to ranibizumab were reported in 3.43% (n = 6) of the patients (**S2 Table**).

## Discussion

LUMINOUS was a 5-year, prospective, observational, multicenter, open-label, single-arm, global study in medical retina that enrolled >30,000 patients across 42 countries to assess the effectiveness and safety of ranibizumab for all approved indications. It was one of the first real-world studies to enroll 297 patients with mCNV with a majority of Caucasians (approximately 88%). The study population consisted mostly of females (treatment-naïve, 80.6% and prior ranibizumab-treated, 65.7%), which is consistent with other studies [5, 18, 19]. The 1-year results show that with ranibizumab treatment, the VA gains from baseline (mean [SD]) were +9.7 letters from 49.5 (±20.5) in the treatment-naïve group and +1.5 letters from 58.5 (±19.8) in prior ranibizumab-treated patients. The VA gains observed in treatment-naïve patients is comparable with the mean VA gains (7.5 letters) observed in the interim subgroup analyses of the real-world, observational, 12-month PACIFIC study (N = 41) conducted in Germany [24]. Ohno-Matsui et al also reported a VA gain of +9.7 letters in their real-world study in Japan [25]. The final VA achieved in both groups were similar (treatment-naïve, 59.1; prior ranibizumab-treated, 59.9). The better baseline VA in the prior ranibizumab-treated group in comparison with treatment-naïve group could probably be due to the efficacy of ranibizumab treatment (patients had already received at least 1 injection before entering LUMINOUS).

When presented by baseline VA category, treatment-naïve patients with the lowest baseline vision (<23 letters and 23–<39 letters) achieved a gain of +18.6 and +21.5 letters, respectively. In the prior ranibizumab-treated group, patients with the lowest baseline vision had greater VA gains (+13.8 letters and +4.6 letters, respectively) when compared with patients having a baseline VA of 39–<60 (+2.9 letters) or 60–<74 letters (+2.6 letters). This could be due to “ceiling effect”. These findings correlate with the other retrospective studies in which patients with lower baseline values had greater VA gains with anti-VEGF treatment [26]. Similar observations were found in the subgroup analyses of the RADIANCE study where the highest VA gain (+20.3 letters) was achieved by patients with the lowest baseline VA of <45 letters [10]. In the present study, the VA losses among the ≥74 letters category were comparable between treatment-naïve and prior ranibizumab-treated groups (−3.4 vs −5.2 letters). The final VA at 1 year was much better in the ≥74 letters category when compared with all the other categories (<74 letters).

In general, the onset of mCNV occurs at an early age and many studies have reported a significant association between the age of diagnosis and VA outcome [9, 27–29]. In this study population, the mean (SD) age of the treatment-naïve patients was 57.6 (±15.6) [range 19–91] years and the prior ranibizumab-treated group was 59.0 (±14.9) [range 19–94] years. Consistent with the literature, both prior ranibizumab-treated and treatment-naïve patients enrolled in the LUMINOUS study were considerably younger when compared to mean age of patients with nAMD. No large difference in age between prior ranibizumab-treated and treatment-naïve patients was observed in the present study.

The mean number of injections over 1 year were 3.0 and 2.6 in the treatment-naïve and prior ranibizumab-treated groups, respectively. However, data was not available to assess if the prior ranibizumab-treated group continued their previous course of treatment or it was a new episode. This is lower than the mean number of injections observed in RCTs such as REPAIR (3.6), RADIANCE (3.5 [ranibizumab treatment guided by disease activity]), and BRILLIANCE (3.9 [ranibizumab treatment guided by disease activity]) [18, 19, 30]. This might be a reflection of under treatment and reduced follow-up, specifically in treatment-naïve patients, which is reflective of any real-world evidence study. However, this gap appeared to be less in mCNV than in nAMD and DME studies [31, 32]. This could also be due to the possibility that patients with mCNV are comparatively younger than patients with nAMD and DME and are more likely to notice any changes in their VA or distortion.

The majority of treatment-naïve and prior ranibizumab-treated patients with mCNV received ≤3 injections during the first year of treatment as observed in the REPAIR and RADIANCE trials which reported greater VA gains with the first three injections in three months [18, 19]. Interestingly, the treatment-naïve patients who received 1–2 injections had marked VA gains at 1 year (+15.0 letters) whereas there was less than 1-letter loss in patients who received ≥5 injections. A clear explanation cannot be made and one can only hypothesize about the possibility of an intervention bias, that patients with less VA gains were treated more intensively. Another aspect might be that patients with a higher refractive error, larger CNV size, and thicker central macular thickness at baseline ended up receiving more injections, probably reflecting a more aggressive type of mCNV. More prospective trials would be needed to understand this phenomenon.

In the country-specific analyses, the mean change in VA and the time from diagnosis to treatment varied between the high enrolling countries in the treatment-naïve patients. Poland and Spain had better VA gains with less time from diagnosis to treatment (Poland, +20.1 letters [baseline VA = 49.5], 4 days; Spain, +12.3 letters [baseline VA = 51.4], 4 days) and Russia had comparatively less BCVA gains with more time to treatment (+3.1 letters [baseline VA = 46.8], 38 days). This suggests that the VA gain is higher if the treatment is initiated earlier. Nevertheless, all conclusions have to be made cautiously due to the relatively low numbers of patients in the country-based analysis. Other factors, such as genetic make-up (majority were Caucasian), possible country-level differences in the time of disease diagnosis or onset, baseline medical comorbidities, less rigorous follow-ups, differences in the health care systems, or reimbursement policies could have contributed to the differences.

It is known, that in addition to baseline VA, the prognostic factors for VA outcome after anti-VEGF therapy in patients with mCNV include age, CNV size and location, presence of chorioretinal atrophy, choroidal thickness, and recurrence of mCNV [26, 28, 33–37]. Long-term follow-up of mCNV is required since mCNV can recur in patients after discontinuing anti-VEGF treatment [38]. An adequate number of injections and follow-up are necessary to avoid any recurrence of mCNV in the long term. Ng *et al*. observed that a longer duration of follow-up was necessary to capture the eyes that required retreatment and the rate of the recurrence of mCNV increased with time [39]. However, the 1-year results of our study does not allow, analysis of these subgroups due to the limited time span.

The strengths of the LUMINOUS study are its observational study design with no strict mandated visit schedule or treatment regimen, which clearly depicts the real-world outcomes in treatment-naïve and prior ranibizumab-treated patients with mCNV. Patients with comorbidities who would be excluded from RCTs were included. Treatment was solely at the investigator’s discretion which reflects the situation in real-world clinical practice. The data from the prior-treated group further adds information regarding the need for further treatment to expect in the longer term, beyond the first year that RCTs have evaluated. To our knowledge, LUMINOUS is one of the largest real-world evidence study in the treatment of mCNV (N = 297), conducted in 42 countries. Additionally, the majority of Caucasians in the study provides an opportunity to understand the treatment outcomes specifically in this population, who overall showed good VA outcomes with fewer injections.

The LUMINOUS study had various limitations. The study had no comparator arm. There could be treatment bias due to the patient’s access to treatment, physicians’ decisions, local healthcare systems and reimbursement policies, which limits some interpretation of data. The lack of a central reading center to evaluate the data collected from the patients and the lack of information on the prognostic markers, such as choroidal thickness and stage of myopic chorioretinal atrophy, could also be considered limiting. There were no strict criteria for disease diagnosis in patient enrollment; hence may present a variable picture. Most of the limitations described are common to any real-world evidence study and are essential aids to collect real-world clinical practice data, which was the intention of this research [40].

To conclude, real-world evidence from the LUMINOUS study in patients with mCNV confirms greater VA gains in treatment-naïve patients and VA maintenance in prior ranibizumab-treated patients at 1 year. The safety findings in the LUMINOUS study were consistent with the well-established safety profile of ranibizumab. No new safety signals were identified. Although retinal detachment is commonly reported in patients with myopia, there was only one incidence in this study [41–43]. To our knowledge, this is the first study with a high patient enrollment diagnosed with mCNV, particularly Caucasians. The results of this study may help ophthalmologists to understand treatment outcomes in real-world clinical practice in mCNV patients and to optimize the treatment and clinical management of the disease. Finally, the study reiterates patient education on regular follow-ups [17].

## Supporting information

S1 Methods (DOCX)

S1 Fig Mean change in VA and mean injection numbers for overall and top four recruited countries (Russia, Poland, UK, and Spain) in treatment-naïve patients with mCNV. Countries with n ≥7 treatment-naïve patients with mCNV with highest evaluable baseline and Month 12 data are included here. The mean number of injections from baseline to Month 12 is designated with a syringe. ETDRS, Early treatment diabetic retinopathy study; mCNV, myopic choroidal neovascularization; n, number of patients; VA, visual acuity.
(TIFF)

S1 Table List of independent ethics committees or institutional review boards. (DOCX)

S2 Table Ocular adverse events suspected to be related to ranibizumab in treatment-naïve and prior ranibizumab-treated patients with mCNV at 1 year. (DOCX)

S3 Table The LUMINOUS study principal investigators. (DOCX)
