# Supplementary material for: Real-world effectiveness and safety of ranibizumab for the treatment of myopic choroidal neovascularization: Results from the LUMINOUS study
Source: PLoS One. 2020 Jan 21;15(1):e0227557. doi: 10.1371/journal.pone.0227557 (PMC6974143; doi:10.1371/journal.pone.0227557)
Supplement: S1 Methods — (DOCX) [file pone.0227557.s001.docx]

**S1 Methods.**

**Treatments**

Enrolled patients were treated with intravitreal ranibizumab 0.5 mg according to the local product label, at outpatient ophthalmology clinics. Since the patients were recruited over time and the calendar time point of study completion was pre-set, follow-up time varied according to the entry dates. The minimum potential follow-up for each patient was defined as 1 year in the protocol. Visits took place at a frequency determined by the investigator. Data from all visits were documented in the electronic case report form (eCRF). It was recommended to capture data in the eCRF at every visit or at a minimum of every 3 months. Physicians were encouraged to follow-up with patients who were not seen in the clinic for at least 6 months since the last visit, in order to capture data. Patients not seen at least once per year, or those switched to another anti-VEGF therapy were discontinued from the study.

Information regarding pre-treatment with ranibizumab or other intravitreal medications was recorded on the prior ocular medications/therapies eCRF page. For each patient, only the data collected over the 365 days after the baseline date (±45 days; Day 319 through Day 409) were included. The time periods were not mutually exclusive, so the patients might have visited at different time periods simultaneously. If so these patients were classified as lost to follow-up in this study.

Treatment-naïve eyes were defined as eyes that have not been pre-treated with any intravitreal medication (ranibizumab, VEGF inhibitor, corticosteroid, excimer refractive laser and verteporfin photodynamic therapy [vPDT] treatment). Eyes previously treated with ranibizumab (treatment non-naïve/ prior-treated) were defined as eyes that have been pre-treated with at least one treatment of ranibizumab regardless of other treatments.

**Assessments and analysis**

VA was recorded using Early Treatment Diabetic Retinopathy Study (ETDRS) letters or Snellen charts. To facilitate data analysis, all Snellen scores were converted to the equivalent ETDRS letter scores. Effectiveness data were presented only for patients for whom baseline and year 1 data were captured. All ranibizumab treatments, VA measurements, ocular adverse events (AEs), and non-ocular AEs were recorded at each patient follow-up visit. The mean change in VA from baseline by injection frequency and baseline VA category were evaluated in the post hoc analyses.
