# Supplementary figures and images for: Real-world effectiveness and safety of ranibizumab for the treatment of myopic choroidal neovascularization: Results from the LUMINOUS study
Source: PLoS One. 2020 Jan 21;15(1):e0227557. doi: 10.1371/journal.pone.0227557 (PMC6974143; doi:10.1371/journal.pone.0227557)

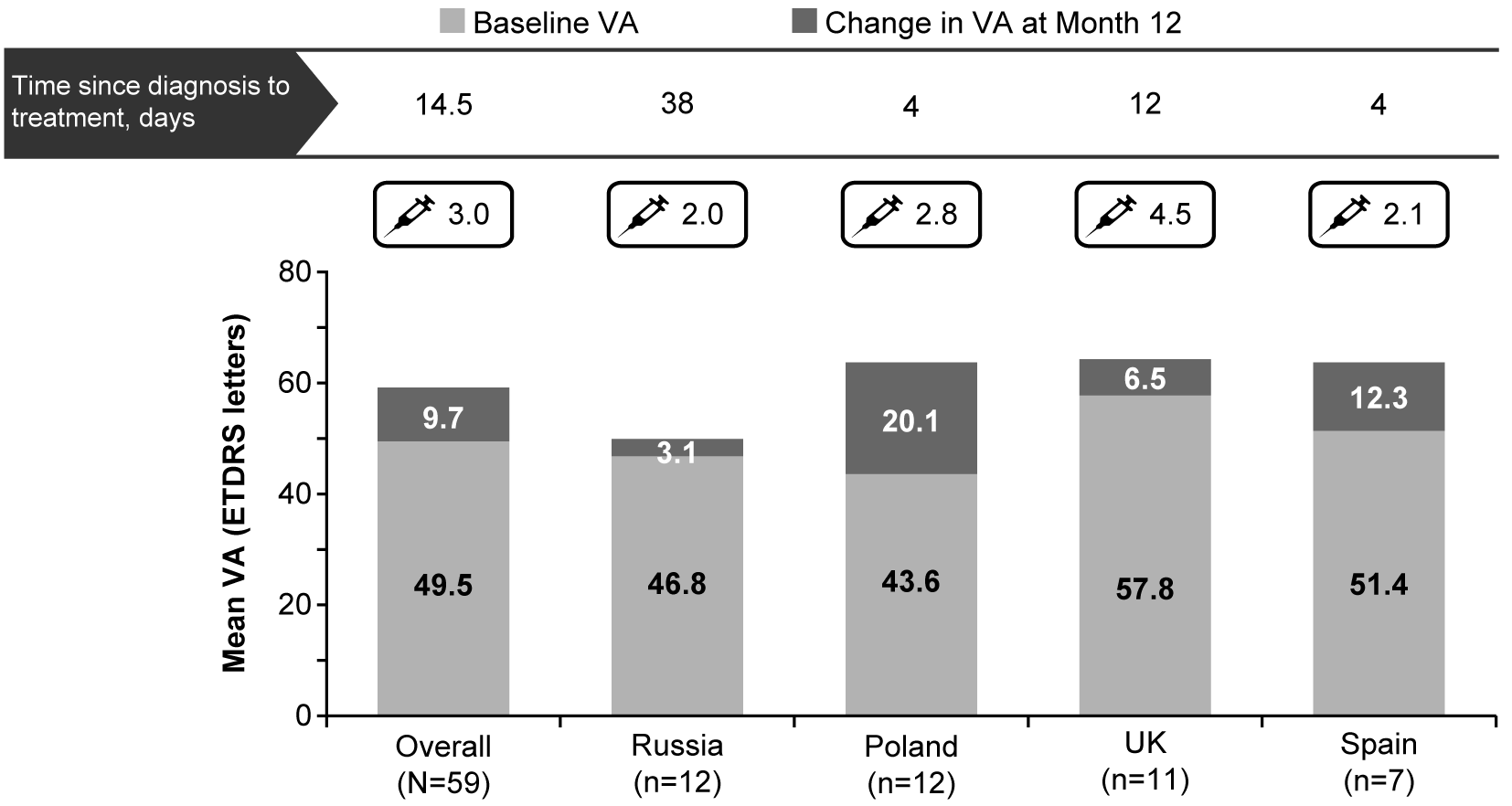

Supplement: S1 Fig — Countries with n ≥7 treatment-naïve patients with mCNV with highest evaluable baseline and Month 12 data are included here. The mean number of injections from baseline to Month 12 is designated with a syringe. ETDRS, Early treatment diabetic retinopathy study; mCNV, myopic choroidal neovascularization; n, number of patients; VA, visual acuity. (TIFF) [file pone.0227557.s002.tiff]
