# Supplementary material for: Real-world effectiveness and safety of ranibizumab for the treatment of myopic choroidal neovascularization: Results from the LUMINOUS study
Source: PLoS One. 2020 Jan 21;15(1):e0227557. doi: 10.1371/journal.pone.0227557 (PMC6974143; doi:10.1371/journal.pone.0227557)
Supplement: S1 Table — (DOCX) [file pone.0227557.s003.docx]

**S1 Table. List of Independent Ethics Committees (IEC) or Institutional Review Boards (IRB) by study center**

| **Center Number** | **Ethics Committee or Institutional Review Board** | **Department / Organization** | **EC/IRB**  **City** | **EC/IRB**  **State/ Province** | **EC/IRB**  **Postal Code** | **Center Country** |
| --- | --- | --- | --- | --- | --- | --- |
| 1100 | Comite Independiente de Etica para Ensayos en Famacologia Clinica | Consultorio de Investigaciones Oftalmológicas | Buenos Aires | Buenos Aires | C1027AAP | Argentina |
| 1101 | Comité de Ética en Investigación Instituto de Investigaciones Clínicas | Clínica Privada de Ojos José León Suárez | Buenos Aires | Buenos Aires | C1027AAP | Argentina |
| 1102 | Comite Independiente de Etica para Ensayos en Famacologia Clinica | Consultorio Dr. Andres Jakofsky | Buenos Aires | Buenos Aires | C1027AAP | Argentina |
| 1103 | Comite Independiente de Etica para Ensayos en Famacologia Clinica | Clínica de Ojos | Buenos Aires | Entre Rios | C1027AAP | Argentina |
| 1104 | Comite Independiente de Etica para Ensayos en Famacologia Clinica | DYTER S.A. | Buenos Aires | Mendoza | C1027AAP | Argentina |
| 1105 | Comite Independiente de Etica para Ensayos en Famacologia Clinica | Clínica de Ojos Srl | Buenos Aires | Santa Fe | C1027AAP | Argentina |
| 1106 | Comite Independiente de Etica para Ensayos en Famacologia Clinica | Microcirugia Ocular (Clínica MICRO) | Buenos Aires | Santa Fe | C1027AAP | Argentina |
| 1107 | Comite Independiente de Etica para Ensayos en Famacologia Clinica | Instituto Donato | Buenos Aires | Ciudad Autonoma Buenos Aires | C1027AAP | Argentina |
| 1108 | Comite Independiente de Etica para Ensayos en Famacologia Clinica | Instituto de la Visión | Buenos Aires | Ciudad Autonoma Buenos Aires | C1027AAP | Argentina |
| 1109 | Comité de Ética en Investigación Instituto de Investigaciones Clínicas | *Oftalmologia Integral* | Mar del Plata | Buenos Aires | B7600FZN | Argentina |
| 1110 | Comité de Ética en Investigación Instituto de Investigaciones Clínicas | Clinica Modelo de Lanus | Buenos Aires | Buenos Aires | C1027AAP | Argentina |
| 1111 | Comite Independiente de Etica para Ensayos en Famacologia Clinica | Centro Oftalmologico Ventola | Buenos Aires | Buenos Aires | C1027AAP | Argentina |
| 1113 | Comite Independiente de Etica para Ensayos en Famacologia Clinica | Clínica Dr Rivero Covre | Buenos Aires | Santa Fe | C1027AAP | Argentina |
| 1114 | Comite Independiente de Etica Fundacion Rusculleda | Instituto Oftamológico de Córdoba SA | Cordoba | Cordoba | X5003DC E | Argentina |
| 1115 | Comite de Etica de CER Investigaciones Clinicas (CECIC) | Hospital Oftalmológico Malvinas Argentinas | Quilmes | Buenos Aires | B1878DVB | Argentina |
| 1116 | Comite Independiente de Etica para Ensayos en Famacologia Clinica | Consultorios Oftalmologico Dres Fugazzotto | Buenos Aires | Mendoza | C1027AAP | Argentina |
| 1117 | Comite Independiente de Etica para Ensayos en Famacologia Clinica | Plaza Vision S.A. | Buenos Aires | Mendoza | C1027AAP | Argentina |

| **Center Number** | **Ethics Committee or Institutional Review Board** | **Department / Organization** | **EC/IRB**  **City** | **EC/IRB**  **State/ Province** | **EC/IRB**  **Postal Code** | **Center Country** |
| --- | --- | --- | --- | --- | --- | --- |
| 1118 | Comite Independiente de Etica para Ensayos en Famacologia Clinica | Grupo laser Visión - Rosario Eximer Laser Visión | Buenos Aires | Santa Fe | C1027AAP | Argentina |
| 1119 | Comite de Etica de CER Investigaciones Clinicas (CECIC) | Clínica Oftalmológica Meroni | Quilmes | Buenos Aires | B1878DVB | Argentina |
| 3001 | Ethik-Kommision Der Medizinischen Universitat Wien | General University Hospital of Vienna | Wien | Vienna | 1090 | Austria |
| 3002 | Ethik-Kommision Der Medizinischen Universitat Wien | Prof. Dr. Siegfried Priglinger | Wien | Vienna | 1090 | Austria |
| 2000 | NSW Government Health Sydney Local Health District | Sydney Eye Hospital, Research Development Office | Camperdo wn | New South Wales | 2050 | Australia |
| 2001 | Bellberry Human Research Ethics Committee | Lions Eye Institute | North Adelaide | South Australia | 5065 | Australia |
| 2002 | Human Research Ethics Committee (Tasmania) Network | Hobart Eye Surgeions, University of Tasmania | Hobart | Tasmania | 7001 | Australia |
| 2003 | Bellberry Human Research Ethics Committee | Eye Consultants SA | North Adelaide | South Australia | 5065 | Australia |
| 2004 | Bellberry Human Research Ethics Committee | Vision Eye Institute Chatswood | North Adelaide | South Australia | 5065 | Australia |
| 2005 | Tasmania Health and Medical Human Research Ethics Committee | Office of Research Services, University of Tasmania | Hobart | Tasmania | 7001 | Australia |
| 2006 | Bellberry Human Research Ethics Committee | Retina & Vitreous Centre | North Adelaide | New South Wales | 5065 | Australia |
| 2007 | Bellberry Human Research Ethics Committee | Vision Retinal Institute | North Adelaide | Queenslan d | 5065 | Australia |
| 2008 | Bellberry Human Research Ethics Committee | Forster Eye Surgery | North Adelaide | New South Wales | 5065 | Australia |
| 2010 | The Royal Victorian Eye & Ear Hospital | Human Research & Ethics Committee | East Melbourne | Victoria | 8002 | Australia |
| 2011 | Bellberry Human Research Ethics Committee | Brisbane Eye Clinic | North Adelaide | Queenslan d | 5065 | Australia |
| 2012 | Bellberry Human Research Ethics Committee | Sydney Retina Eye Clinic and Day Surgery | North Adelaide | New South Wales | 5065 | Australia |
| 2013 | Bellberry Human Research Ethics Committee | Macquarie University | North Adelaide | New South Wales | 5064 | Australia |
| 2014 | Bellberry Human Research Ethics Committee | Marsden Eye Specialists | North Adelaide | New South Wales | 5065 | Australia |
| 2017 | Bellberry Human Research Ethics Committee | Southern Ophthalmology | North Adelaide | New South Wales | 5065 | Australia |
| 2018 | Bellberry Human Research Ethics Committee | Strathfield Retina Clinic | North Adelaide | New South Wales | 5065 | Australia |
| 2019 | Bellberry Human Research Ethics Committee | Retina Associates - Chatswood Retina Service | North Adelaide | New South Wales | 5065 | Australia |
| 2020 | Bellberry Human Research Ethics Committee | Macquarie University | North Adelaide | New South Wales | 5065 | Australia |
| 2021 | Bellberry Human Research Ethics Committee | Adelaide Eye and Retina Centre | North Adelaide | South Australia | 5065 | Australia |
| 2024 | Bellberry Human Research Ethics Committee | Queensland Eye Institute | North Adelaide | Queenslan d | 5065 | Australia |

| **Center Number** | **Ethics Committee or Institutional Review Board** | **Department / Organization** | **EC/IRB**  **City** | **EC/IRB**  **State/ Province** | **EC/IRB**  **Postal Code** | **Center Country** |
| --- | --- | --- | --- | --- | --- | --- |
| 2025 | Bellberry Human Research Ethics Committee | Melbourne Retina Associates | North Adelaide | Victoria | 5065 | Australia |
| 2026 | Bellberry Human Research Ethics Committee | Private Rooms, Eye Clinic (B4a) Westmead Hospital | North Adelaide | New South Wales | 5065 | Australia |
| 2032 | Bellberry Human Research Ethics Committee | St. John of God Hospital | North Adelaide | Victoria | 5065 | Australia |
| 2034 | Bellberry Human Research Ethics Committee | Oakleigh Eye Center | North Adelaide | Victoria | 5065 | Australia |
| 2035 | Bellberry Human Research Ethics Committee | Retina and Vitreous Centre | North Adelaide | New South Wales | 5065 | Australia |
| 2037 | Bellberry Human Research Ethics Committee | Waverley Eye Clinic | North Adelaide | Victoria | 5065 | Australia |
| 3100 | Committee for Medical Ethics/Clinical Research | Faculty of Medicine UZ Gathuisberg | Leuven |  | 3000 | Belgium |
| 3101 | Committee for Medical Ethics/Clinical Research | Faculty of Medicine UZ Gathuisberg | Leuven | Hasselt | 3000 | Belgium |
| 3102 | Committee for Medical Ethics/Clinical Research | Faculty of Medicine UZ Gathuisberg | Leuven |  | 3000 | Belgium |
| 3104 | Committee for Medical Ethics/Clinical Research | Faculty of Medicine UZ Gathuisberg | Leuven |  | 3000 | Belgium |
| 3106 | Committee for Medical Ethics/Clinical Research | Faculty of Medicine UZ Gathuisberg | Leuven |  | 3000 | Belgium |
| 3107 | Committee for Medical Ethics/Clinical Research | Faculty of Medicine UZ Gathuisberg | Leuven |  | 3000 | Belgium |
| 3110 | Committee for Medical Ethics/Clinical Research | Faculty of Medicine UZ Gathuisberg | Leuven |  | 3000 | Belgium |
| 3111 | Committee for Medical Ethics/Clinical Research | Faculty of Medicine UZ Gathuisberg | Leuven |  | 3000 | Belgium |
| 3112 | Committee for Medical Ethics/Clinical Research | Faculty of Medicine UZ Gathuisberg | Leuven |  | 3000 | Belgium |
| 3113 | Comite d'Ethique Centre Hospitalier de Mouscron | Centre Hospitalier de Mouscron | Mouscron |  | 7700 | Belgium |
| 3114 | Committee for Medical Ethics/Clinical Research | Faculty of Medicine UZ Gathuisberg | Leuven |  | 3000 | Belgium |
| 3117 | Committee for Medical Ethics/Clinical Research | Faculty of Medicine UZ Gathuisberg | Leuven |  | 3000 | Belgium |
| 3118 | Committee for Medical Ethics/Clinical Research | Faculty of Medicine UZ Gathuisberg | Leuven |  | 3000 | Belgium |
| 3120 | Committee for Medical Ethics/Clinical Research | Faculty of Medicine UZ Gathuisberg | Leuven |  | 3000 | Belgium |
| 1200 | Hospital Municipal Dr. Mario Gatti | Centro Medico de Oftalmologia | Cambui | Campinas | 13092 | Brazil |
| 1203 | Comissao Nacional de Etica Em Pesquisa | Instituto da Visão | Belo Horizonte | Minas Gerais | 30150 | Brazil |
| 1204 | Comissao Nacional de Etica Em Pesquisa | HFSE - Hospital Federal dos Servidores do Estado do Rio de Janeiro | Belo Horizonte | Rio de Janeiro | 30150 | Brazil |
| 1205 | Comissao Nacional de Etica Em Pesquisa | CBCO - Centro Brasileiro de Cirurgia de Olhos | Belo Horizonte | Goiás | 30150 | Brazil |
| 1208 | Comissao Nacional de Etica Em Pesquisa | ANGIOCORPORE | Belo Horizonte | Sao Paulo | 30150 | Brazil |
| 1209 | Comissao Nacional de Etica Em Pesquisa | Clínica Lavinsky Oftalmologia | Belo Horizonte | Rio Grande do Sul | 30150 | Brazil |
| 1000 | Ontario IRB/REB | IRB Services | Aurora | New Brunswick | L4G 0A5 | Canada |

| **Center Number** | **Ethics Committee or Institutional Review Board** | **Department / Organization** | **EC/IRB**  **City** | **EC/IRB**  **State/ Province** | **EC/IRB**  **Postal Code** | **Center Country** |
| --- | --- | --- | --- | --- | --- | --- |
| 1001 | Ontario IRB/REB | IRB Services | Aurora | Nova Scotia | L4G 0A5 | Canada |
| 1003 | Ontario IRB/REB | IRB Services | Aurora | Ontario | L4G 0A5 | Canada |
| 1008 | Ontario IRB/REB | IRB Services | Aurora | Ontario | L4G 0A5 | Canada |
| 1007 | University Health Network | Research Ethics Board | Toronto | Ontario | M5G 1Z5 | Canada |
| 1006 | Ontario IRB/REB | IRB Services | Aurora | Ontario | L4G 0A5 | Canada |
| 1005 | Ontario IRB/REB | IRB Services | Aurora | Ontario | L4G 0A5 | Canada |
| 1004 | Ontario IRB/REB | IRB Services | Aurora | Quebec | L4G 0A5 | Canada |
| 1010 | Ontario IRB/REB | IRB Services | Aurora | Quebec | L4G 0A5 | Canada |
| 1011 | Ottawa Health Science Network Research Ethics Board | The Ottawa Hospital  - General Campus, University of Ottawa Eye Institute | Ottawa | Ontario | K1Y 4E9 | Canada |
| 1012 | Ontario IRB/REB | The Retina Centre of Ottawa | Aurora | Ontario | L4G 0A5 | Canada |
| 1013 | Ontario IRB/REB | IRB Services | Aurora | Ontario | L4G 0A5 | Canada |
| 1014 | Ontario IRB/REB | IRB Services | Aurora | British Columbia | L4G 0A5 | Canada |
| 1015 | Health Research Ethics Board of Alberta | IRB Services | Edmonton | Alberta | T5J 4A7 | Canada |
| 1016 | Ontario IRB/REB | Clincial Trials Committee | Aurora | Ontario | L4G 0A5 | Canada |
| 1018 | Ontario IRB/REB | IRB Services | Aurora | Ontario | L4G 0A5 | Canada |
| 1020 | Ontario IRB/REB | IRB Services | Aurora | Ontario | L4G 0A5 | Canada |
| 1021 | Ontario IRB/REB | IRB Services | Aurora | Ontario | L4G 0A5 | Canada |
| 1019 | Ontario IRB/REB | IRB Services | Aurora | Ontario | L4G 0A5 | Canada |
| 8200 | Resolucion Comite Etico Cientifico | Hospital del Cobre Salvador Allende Gossens | La Serena |  | 1399001 | Chile |
| 2101 | Beijing Tongren Hospital EC | Renmin Hospital of Wuhan University | Wuhan | Hubei | 430060 | China |
| 2102 | Beijing Tongren Hospital EC | No. 10 People's Hospital of Shanghai |  | Shanghai | 200072 | China |
| 2104 | Beijing Tongren Hospital EC | Eye and ENT hospital |  | Shanghai | 200000 | China |
| 2106 | Beijing Tongren Hospital EC | Zhongshan Ophthalmic Center, Sun Yat-sen University | Guangzho u | Guangdon g | 510060 | China |
| 2107 | Beijing Tongren Hospital EC | Peking University First Hospital | Beijing,P. R. | Beijing | 100034 | China |
| 2108 | Beijing Tongren Hospital EC | Tianjin Medical University Eye Center | Tianjin |  | 300384 | China |
| 2109 | Beijing Tongren Hospital EC | Xinhua Hospital Affiliated to Shanghai Jiao Tong University School of Medicine |  | Shanghai | 200092 | China |
| 2100 | Beijing Tongren Hospital EC | Beijing Tong Ren Hospital, Capital Medical University |  | Beijing | 100730 | China |
| 2115 | Beijing Tongren Hospital EC | Peking University Third Hospital |  | Beijing | 100191 | China |
| 2116 | Beijing Tongren Hospital EC | Chinese PLA General Hospital | Beijing | Beijing | 100853 | China |
| 2117 | Beijing Tongren Hospital EC | Beijing Hospital |  | Beijing | 100730 | China |
| 2119 | Beijing Tongren Hospital EC | Peking Union Medical College Hospital |  | Beijing | 100032 | China |
| 2121 | Beijing Tongren Hospital EC | Southwest Hospital | Chongqing | Chongqing | 400038 | China |
| 2122 | Beijing Tongren Hospital EC | Shanghai First People's Hospital |  | Shangai | 200080 | China |
| 2124 | Beijing Tongren Hospital EC | Xiamen Eye Centre | Fujian |  | 361001 | China |

| **Center Number** | **Ethics Committee or Institutional Review Board** | **Department / Organization** | **EC/IRB**  **City** | **EC/IRB**  **State/ Province** | **EC/IRB**  **Postal Code** | **Center Country** |
| --- | --- | --- | --- | --- | --- | --- |
| 2127 | Beijing Tongren Hospital EC | Shierming Eye Hospital | Jinan | Shandong | 250001 | China |
| 2128 | Beijing Tongren Hospital EC | The Affiliated Hospital of Guiyang Medical College | Guiyang | Guizhou | 500000 | China |
| 2126 | Beijing Tongren Hospital EC | No. 474 Hospital of PLA | Wulumuqi | Xinjiang Uygur | 830000 | China |
| 2130 | Beijing Tongren Hospital EC | No.2 Hospital Affiliated to Jilin University | Changchu n City | Jilin | 130041 | China |
| 2129 | Beijing Tongren Hospital EC | Yunnan 2nd People's Hospital | Kunming | Yun'nan | 650021 | China |
| 1304 | Sociedad de Cirugia Ocular S.A. | Sociedad de Cirugía Ocular | Bogota |  |  | Colombia |
| 1302 | Instituto para Ninos Ciegos y Sordos del Valle del Cauca | Instituto de Ciegos y Sordos INSORP | San Fernando | Cali |  | Colombia |
| 1306 | Sociedad de Cirugia Ocular S.A. | Clínica Barraquer | Bogota |  |  | Colombia |
| 1307 | Sociedad de Cirugia Ocular S.A. | Clinica Oftalmologica | Bogota |  |  | Colombia |
| 1309 | Comite de Etica en la Investigacion | OPTISALUD SAS | Bogota |  |  | Colombia |
| 1308 | Sociedad de Cirugia Ocular S.A. | Clínica Oftalmológica del Atlántico | Bogota |  |  | Colombia |
| 3300 | Eticka komise Fakultni nemocnice Hradec Kralove | Fakultni nemocnice Hradec Kralove | Hradec Kralove |  | 500 05 | Czech Republic |
| 3302 | Eticka komise Fakultni Nemocnice Kralovske Vinohrady | Fakultni nemocnice Kralovske Vinohrady | Praha |  | 100 34 | Czech Republic |
| 3303 | Eticka komise FN Ostrava | University Hopsital Ostrava | Ostrava- Poruba |  | 70800 | Czech Republic |
| 3305 | Eticka komise Vseobecne fakultni nemocnice v Praze | Charles University Hopsital 1st Faculty of Medicine | Praha 2 | Praha 2 | 128 08 | Czech Republic |
| 3307 | Eticka komise Fakultni Nemocnice Brno | Fakultni nemocnice Brno | Brno |  | 625 00 | Czech Republic |
| 3306 | Eticka komise Ustredni vojenske nemocnice | Ustredni vojenska nemocnice Praha | Praha 6 |  | 169 02 | Czech Republic |
| 3301 | Eticka komise-Krajska zdravotni a.s. | Krajska zdravotni,  a.s. - Masarykova nemocnice v Usti nad Labem | Usti nad Labem |  | 40113 | Czech Republic |
| 3308 | Eticka komise FN a LF UP Olomouc-LEC | Fakultni nemocnice Olomouc | Olomouc |  | 775 20 | Czech Republic |
| 3304 | Eticka komise Fakultni nemocnice Plzen | Fakultni nemocnice Plzen | Plzen |  | 305 99 | Czech Republic |
| 3600 | Ethikkommission an der Medizinischen Fakultaet der Rheinischen Friedrich-Wilhelms- Universitaet Bonn | University of Bonn | Bonn | Nordrhein Westfalen | 53105 | Germany |
| 3601 | Aerztekammer Berlin | Praxis Arzt für Augenheilkunde | Berlin |  | 10969 | Germany |
| 3604 | Ethik-Kommission der Ärztekammer Westfalen- Lippe und der Med.  Fakultät der Universität Münster | St. Franziskus Hospital | Muenster | Nordrhein Westfalen | 48147 | Germany |
| 3602 | Ethikkommission an der Medizinischen Fakultaet der Rheinischen Friedrich-Wilhelms- Universitaet Bonn | Augenarztpraxis Grasbon | Bonn | Bayern | 53105 | Germany |
| 3606 | Aerztekammer Berlin | Praxis Steinberg | Berlin |  | 10969 | Germany |
| 3607 | Ethik-Kommission der Ärztekammer Westfalen- Lippe und der Med.  Fakultät der Universität Münster | Universitaetsklinikum Muenster | Muenster | Nordrhein Westfalen | 48147 | Germany |

| **Center Number** | **Ethics Committee or Institutional Review Board** | **Department / Organization** | **EC/IRB**  **City** | **EC/IRB**  **State/ Province** | **EC/IRB**  **Postal Code** | **Center Country** |
| --- | --- | --- | --- | --- | --- | --- |
| 3608 | Ethikkommission der erztekammer Hamburg | Praxis_Dr Kaupke | Hamburg |  | 22083 | Germany |
| 3609 | Ethik-Kommission der Ärztekammer Westfalen- Lippe und der Med.  Fakultät der Universität Münster | Klinikum Lüdenscheid | Muenster | Nordrhein Westfalen | 48147 | Germany |
| 3611 | Landesaerztekammer Baden-Wuerttemberg | Dr. Rabethge Klinik GmbH | Stuttgart | Baden Wuerttem berg | 70597 | Germany |
| 3612 | Universitaetsklinikum Tuebingen | Eberhard Karls University Eye Hospital | Tuebingen | Baden Wuerttem berg | 72074 | Germany |
| 3615 | Ethik-Kommission der Aerztekammer Niedersachsen | Klinikum Osnabrück | Hannover | Niedersac hsen | 30175 | Germany |
| 3616 | Geschaeftsstelle der Ethikkommission | Universitaetsklinikum Koeln | Koeln | Nordrhein Westfalen | 50931 | Germany |
| 3628 | Augenlaserzentrum Neu- Ulm | Augenlaserzentrum | Neu-Ulm | Bayern | 89231 | Germany |
| 3629 | Ethik-Kommission der Aerztekammer Niedersachsen | Augenklinik Dannenberg | Hannover | Niedersac hsen | 30175 | Germany |
| 3632 | Ethik-Kommission der Ärztekammer Westfalen- Lippe und der Westfälischen Wilhelms- Universität Münster | Private practice_ Dr Grote-Schmidt | Münster | Nordrhein Westfalen | 48147 | Germany |
| 3631 | An die Ethik-Kommission der Bayerischen Landesärztekammer | Fachärztin für Augenheilkunde | Munich | Bayern | 81677 | Germany |
| 3633 | An die Ethik-Kommission der Bayerischen Landesärztekammer | Klinikum Augsburg Augenklinik | Munich | Bayern | 81677 | Germany |
| 3630 | An die Ethik-Kommission der Bayerischen Landesärztekammer | Dr. med. Claus Fuchs Fachärzte für Augenheilkunde | Munich | Bayern | 81677 | Germany |
| 8300 | Comite de Bioetica de la Universidad Central del Ecuador (COBI-UCE) | Centro Medico Quirurgico Oftalmologico Alta Vision | Quito |  | 593 | Ecuador |
| 4600 | CEIC Hospital Universitario Ramon y Cajal | Vissum Corporación Oftalmológica – Mirassierra | Madrid |  | 28034 | Spain |
| 4601 | CEIC Hospital Universitario Ramon y Cajal | Hospital Clinico Universitario Lozano Blesa | Madrid |  | 28035 | Spain |
| 4602 | CEIC Hospital Universitario Ramon y Cajal | Hospital Universitari i Politecnic La Fe | Madrid |  | 28036 | Spain |
| 4604 | CEIC Hospital Universitario Ramon y Cajal | Hospital Universitario Clinico San Carlos | Madrid |  | 28039 | Spain |
| 4605 | CEIC Hospital Universitario Ramon y Cajal | Hospital Universitario 12 de Octubre | Madrid |  | 28040 | Spain |
| 4606 | CEIC Hospital Universitario Ramon y Cajal | Hospital Universitario Miguel Servet | Madrid |  | 28043 | Spain |
| 4608 | CEIC Hospital Universitario Ramon y Cajal | Hospital de la Santa Creu i Sant Pau | Madrid |  | 28045 | Spain |
| 4612 | CEIC Hospital Universitario Ramon y Cajal | Universidad de Valladolid | Madrid |  | 28049 | Spain |
| 4614 | CEIC Hospital Universitario Ramon y Cajal | Hospital General de Catalunya | Madrid | Barcelona | 28049 | Spain |

| **Center Number** | **Ethics Committee or Institutional Review Board** | **Department / Organization** | **EC/IRB**  **City** | **EC/IRB**  **State/ Province** | **EC/IRB**  **Postal Code** | **Center Country** |
| --- | --- | --- | --- | --- | --- | --- |
| 4615 | CEIC Hospital Universitario Ramon y Cajal | Hospital San Pedro | Madrid | La Rioja | 28049 | Spain |
| 4620 | CEIC Hospital Universitario Ramon y Cajal | Hospital Universitario Principe de Asturias | Madrid | Madrid | 28049 | Spain |
| 4622 | CEIC Hospital Universitario Ramon y Cajal | Hospital Universitario de Burgos | Madrid |  | 28049 | Spain |
| 4623 | CEIC Hospital Universitario Ramon y Cajal | Hospital Universitario de Salamanca | Madrid |  | 28049 | Spain |
| 4624 | CEIC Hospital Universitario Ramon y Cajal | Hospital Moncloa | Madrid |  | 28049 | Spain |
| 4625 | CEIC Hospital Universitario Ramon y Cajal | Hospital Universitario de Leon | Madrid |  | 28049 | Spain |
| 4626 | CEIC Hospital Universitario Ramon y Cajal | Hospital Universitario Lucus Augusti | Madrid |  | 28049 | Spain |
| 4627 | CEIC Hospital Universitario Ramon y Cajal | Hospital Dos de Maig | Madrid | Barcelona | 28050 | Spain |
| 4628 | CEIC Hospital Universitario Ramon y Cajal | Hospital Arnau de Vilanova | Madrid |  | 28050 | Spain |
| 4630 | CEIC Hospital Universitario Ramon y Cajal | Clinica Rementeria | Madrid |  | 28051 | Spain |
| 4634 | CEIC Hospital Universitario Ramon y Cajal | Centro de Oftalmología Barraquer | Madrid |  | 28052 | Spain |
| 4632 | CEIC Hospital Universitario Ramon y Cajal | Hospital Regional Universitario de Malaga | Madrid |  | 28053 | Spain |
| 4631 | CEIC Hospital Universitario Ramon y Cajal | Hospital Universitario Sant Joan de Reus | Madrid | Tarragona | 28054 | Spain |
| 4629 | CEIC Hospital Universitario Ramon y Cajal | Fundacio Privada Hospital Asil de Granollers | Madrid | Barcelona | 28055 | Spain |
| 4633 | CEIC Hospital Universitario Ramon y Cajal | Centro Oftalmologico Gaztambide | Madrid |  | 28056 | Spain |
| 3500 | N/A | Centre Hospitalier Intercommunal de Créteil | Creteil | Val de Marne |  | France |
| 3501 | N/A | Centre Ophtalmologique d’Imagerie et de Laser | Paris | Val de Marne | 94010 | France |
| 3502 | N/A | Clinique Ocean | Vannes | Côte-d'Or | 75015 | France |
| 3503 | N/A | CHU Dijon - Hopital General | Dijon | Côte-d'Or | 56000 | France |
| 3504 | N/A | Visiopole Private Practice | Lagord | Charente | 21000 | France |
| 3505 | N/A | CHU de Nice - Hôpital Lenval | Nice | Alpes Maritimes | 17140 | France |
| 3506 | N/A | Cabinet Odeon | Paris |  | 060 06 | France |
| 3507 | N/A | Groupe Hospitalier Pellegrin -Hôpital Pellegrin | Bordeaux | Gironde | 75006 | France |
| 3518 | N/A | Centre Ophtalmologique de L'Odeon | Paris |  | 33000 | France |
| 3508 | N/A | Hôpital Lariboisière | Paris |  | 75006 | France |
| 3509 | N/A | Polyclinique de Courlancy | Reims | Marne | 75475 | France |
| 3510 | N/A | Clinique Orl Honore Cave | Montauba n | Tarn et Garonne | 51100 | France |
| 3511 | N/A | Cabinet d'Ophtalmologie | Melun | Seine et Marne | 82000 | France |

| **Center Number** | **Ethics Committee or Institutional Review Board** | **Department / Organization** | **EC/IRB**  **City** | **EC/IRB**  **State/ Province** | **EC/IRB**  **Postal Code** | **Center Country** |
| --- | --- | --- | --- | --- | --- | --- |
| 3512 | N/A | CHU Nantes - Hôtel Dieu | Nantes Cedex 1 | Loire Atlantique | 77000 | France |
| 3513 | N/A | Centre Hospitalier de la Croix Rousse | Lyon | Rhone | 44093 | France |
| 3514 | N/A | CHU Toulouse, Hôpital Paule de Vignier | Toulouse | Cedex 9 | 69317 | France |
| 3516 | N/A | Fondation Ophtalmologique Adolphe de Rothschild | Paris |  | 31059 | France |
| 3517 | N/A | Clinique de Montargis | Montargis | Loiret | 75019 | France |
| 5000 | NRES Committee Yorkshire and the Humber Leeds East | Central Ethics Committee | Jarrow | Lancashir e | 45200 | United Kingdom |
| 5001 | NRES Committee Yorkshire and the Humber Leeds East | Central Ethics Committee | Jarrow | South Yorkshire | NE 32 3DT | United Kingdom |
| 5002 | NRES Committee Yorkshire and the Humber Leeds East | Central Ethics Committee | Jarrow | Tyne & Wear | NE 32 3DT | United Kingdom |
| 5003 | NRES Committee Yorkshire and the Humber Leeds East | Central Ethics Committee | Jarrow |  | NE 32 3DT | United Kingdom |
| 5004 | NRES Committee Yorkshire and the Humber Leeds East | Central Ethics Committee | Jarrow | Surrey | NE 32 3DT | United Kingdom |
| 5005 | NRES Committee Yorkshire and the Humber Leeds East | Central Ethics Committee | Jarrow | North Yorkshire | NE 32 3DT | United Kingdom |
| 5006 | NRES Committee Yorkshire and the Humber Leeds East | Central Ethics Committee | Jarrow | Greater Manchest er | NE 32 3DT | United Kingdom |
| 5009 | NRES Committee Yorkshire and the Humber Leeds East | Central Ethics Committee | Jarrow |  | NE 32 3DT | United Kingdom |
| 5030 | NRES Committee Yorkshire and the Humber Leeds East | Central Ethics Committee | Jarrow | Greater London | NE 32 3DT | United Kingdom |
| 5010 | NRES Committee Yorkshire and the Humber Leeds East | Central Ethics Committee | Jarrow | Norfolk | NE 32 3DT | United Kingdom |
| 5011 | NRES Committee Yorkshire and the Humber Leeds East | Central Ethics Committee | Jarrow | Staffordshi re | NE 32 3DT | United Kingdom |
| 5012 | NRES Committee Yorkshire and the Humber Leeds East | Central Ethics Committee | Jarrow |  | NE 32 3DT | United Kingdom |
| 5013 | NRES Committee Yorkshire and the Humber Leeds East | Central Ethics Committee | Jarrow | West Midlands | NE 32 3DT | United Kingdom |
| 5014 | NRES Committee Yorkshire and the Humber Leeds East | Central Ethics Committee | Jarrow | West Midlands | NE 32 3DT | United Kingdom |
| 5015 | NRES Committee Yorkshire and the Humber Leeds East | Central Ethics Committee | Jarrow | West Yorkshire | NE 32 3DT | United Kingdom |
| 5024 | NRES Committee Yorkshire and the Humber Leeds East | Central Ethics Committee | Jarrow | Surrey | NE 32 3DT | United Kingdom |
| 5055 | NRES Committee Yorkshire and the Humber Leeds East | Central Ethics Committee | Jarrow | Greater London | NE 32 3DT | United Kingdom |
| 5036 | NRES Committee Yorkshire and the Humber Leeds East | Central Ethics Committee | Jarrow | Gloucester shire | NE 32 3DT | United Kingdom |
| 5037 | NRES Committee Yorkshire and the Humber Leeds East | Central Ethics Committee | Jarrow | Greater London | NE 32 3DT | United Kingdom |
| 5016 | NRES Committee Yorkshire and the Humber Leeds East | Central Ethics Committee | Jarrow | West Midlands | NE 32 3DT | United Kingdom |

| **Center Number** | **Ethics Committee or Institutional Review Board** | **Department / Organization** | **EC/IRB**  **City** | **EC/IRB**  **State/ Province** | **EC/IRB**  **Postal Code** | **Center Country** |
| --- | --- | --- | --- | --- | --- | --- |
| 5029 | NRES Committee Yorkshire and the Humber Leeds East | Central Ethics Committee | Jarrow | Hampshire | NE 32 3DT | United Kingdom |
| 5021 | NRES Committee Yorkshire and the Humber Leeds East | Central Ethics Committee | Jarrow |  | NE 32 3DT | United Kingdom |
| 5017 | NRES Committee Yorkshire and the Humber Leeds East | Central Ethics Committee | Jarrow | Hampshire | NE 32 3DT | United Kingdom |
| 5022 | NRES Committee Yorkshire and the Humber Leeds East | Central Ethics Committee | Jarrow | Cheshire | NE 32 3DT | United Kingdom |
| 5018 | NRES Committee Yorkshire and the Humber Leeds East | Central Ethics Committee | Jarrow | Greater London | NE 32 3DT | United Kingdom |
| 5019 | NRES Committee Yorkshire and the Humber Leeds East | Central Ethics Committee | Jarrow | North Yorkshire | NE 32 3DT | United Kingdom |
| 5020 | NRES Committee Yorkshire and the Humber Leeds East | Central Ethics Committee | Jarrow | North Yorkshire | NE 32 3DT | United Kingdom |
| 5026 | NRES Committee Yorkshire and the Humber Leeds East | Central Ethics Committee | Jarrow | West Midlands | NE 32 3DT | United Kingdom |
| 5025 | NRES Committee Yorkshire and the Humber Leeds East | Central Ethics Committee | Jarrow | West Midlands | NE 32 3DT | United Kingdom |
| 5044 | NRES Committee Yorkshire and the Humber Leeds East | Central Ethics Committee | Jarrow | Leicesters hire | NE 32 3DT | United Kingdom |
| 5031 | NRES Committee Yorkshire and the Humber Leeds East | Central Ethics Committee | Jarrow | Isle of Wight | NE 32 3DT | United Kingdom |
| 5032 | NRES Committee Yorkshire and the Humber Leeds East | Central Ethics Committee | Jarrow | Lancashir e | NE 32 3DT | United Kingdom |
| 5023 | NRES Committee Yorkshire and the Humber Leeds East | Central Ethics Committee | Jarrow | West Yorkshire | NE 32 3DT | United Kingdom |
| 5028 | NRES Committee Yorkshire and the Humber Leeds East | Central Ethics Committee | Jarrow | Greater London | NE 32 3DT | United Kingdom |
| 5033 | NRES Committee Yorkshire and the Humber Leeds East | Central Ethics Committee | Jarrow | Greater London | NE 32 3DT | United Kingdom |
| 5038 | NRES Committee Yorkshire and the Humber Leeds East | Central Ethics Committee | Jarrow | Oxfordshir e | NE 32 3DT | United Kingdom |
| 5027 | NRES Committee Yorkshire and the Humber Leeds East | Central Ethics Committee | Jarrow | Essex | NE 32 3DT | United Kingdom |
| 5039 | NRES Committee Yorkshire and the Humber Leeds East | Central Ethics Committee | Jarrow | Greater London | NE 32 3DT | United Kingdom |
| 5034 | NRES Committee Yorkshire and the Humber Leeds East | Central Ethics Committee | Jarrow | Merseysid e | NE 32 3DT | United Kingdom |
| 5040 | NRES Committee Yorkshire and the Humber Leeds East | Central Ethics Committee | Jarrow | Devon | NE 32 3DT | United Kingdom |
| 5041 | NRES Committee Yorkshire and the Humber Leeds East | Central Ethics Committee | Jarrow | Highland Region | NE 32 3DT | United Kingdom |
| 5042 | NRES Committee Yorkshire and the Humber Leeds East | Central Ethics Committee | Jarrow |  | NE 32 3DT | United Kingdom |
| 5047 | NRES Committee Yorkshire and the Humber Leeds East | Central Ethics Committee | Jarrow | Norfolk | NE 32 3DT | United Kingdom |
| 5035 | NRES Committee Yorkshire and the Humber Leeds East | Central Ethics Committee | Jarrow | Avon | NE 32 3DT | United Kingdom |

| **Center Number** | **Ethics Committee or Institutional Review Board** | **Department / Organization** | **EC/IRB**  **City** | **EC/IRB**  **State/ Province** | **EC/IRB**  **Postal Code** | **Center Country** |
| --- | --- | --- | --- | --- | --- | --- |
| 5043 | NRES Committee Yorkshire and the Humber Leeds East | Central Ethics Committee | Jarrow | Kent | NE 32 3DT | United Kingdom |
| 5046 | NRES Committee Yorkshire and the Humber Leeds East | Central Ethics Committee | Jarrow | Derbyshire | NE 32 3DT | United Kingdom |
| 5060 | NRES Committee Yorkshire and the Humber Leeds East | Central Ethics Committee | Jarrow | Bedfordshi re | NE 32 3DT | United Kingdom |
| 5061 | NRES Committee Yorkshire and the Humber Leeds East | Central Ethics Committee | Jarrow | Merseysid e | NE 32 3DT | United Kingdom |
| 5062 | NRES Committee Yorkshire and the Humber Leeds East | Central Ethics Committee | Jarrow | Worcester shire | NE 32 3DT | United Kingdom |
| 3700 | Scientific Council of University Eye Hospital of Haraklion | University Eye Hospital of Heraklion | Heraklion |  | 71201 | Greece |
| 3701 | Scientific Council of Eye Hospital of Athens | Eye Hospital of Athens -1st Clinic | Athens |  | 10672 | Greece |
| 3702 | Scientific Council of Omma Opthalmological Institute of Athens | Omma Ophtalmological Institute of Athens | Athens |  | 11525 | Greece |
| 3703 | Scientific Council of General Hospital Papageorgiou | General Hospital Papageorgiou | Thessaloni ki |  | 56429 | Greece |
| 3704 | Scientific Council of Red Cross Hospital | Red Cross Hospital - 2nd Clinic | Athens |  | 11526 | Greece |
| 3705 | Scientific Council of University Hospital of Alexandroupolis | University General Hospital of Alexandroupolis | Alexandro upolis |  | 68100 | Greece |
| 3710 | Ethics Committee of University Hospital of Larissa | University General Hospital of Larissa | Mezourlo |  | 41110 | Greece |
| 3712 | Scientific Council of Athens Vision Eye Institute | Athens Vision Eye Institute | Athens |  | 17673 | Greece |
| 3714 | Scientific Council of Athens Eye Hospital | Ophtalmiatrio Athinon - Athens Eye Hospital | Athens |  | 10672 | Greece |
| 3715 | Ethics Committee of General Hospital of Lamia | General Hospital of Lamia | Lamia |  | 35100 | Greece |
| 3716 | Ethics Committee of University Hospital of Patra | University Hopsital of Patras | Patra |  | 26504 | Greece |
| 3717 | Ethics Committee of General Hospital of Rethymnon | General Hospital of Rethymnon | Rethymno n |  | 74100 | Greece |
| 8800 | Research Ethics Committee (Kowloon Central/Kowloon East) | Hong Kong Eye Hospital | Kowloon |  | N/A | Hong Kong |
| 3800 | Egeszsegugyi Tudomanyos Tanacs Tudomanyos es Kutatasetikai Bizottsag | Budapest Retina Associates | Budapest |  | 1051 | Hungary |
| 3802 | Egeszsegugyi Tudomanyos Tanacs Tudomanyos es Kutatasetikai Bizottsag | Semmelweis Egyetem | Budapest |  | 1051 | Hungary |
| 3801 | Egeszsegugyi Tudomanyos Tanacs Tudomanyos es Kutatasetikai Bizottsag | Debreceni Egyetem Klinikai Kozpont | Budapest |  | 1051 | Hungary |
| 3803 | Egeszsegugyi Tudomanyos Tanacs Tudomanyos es Kutatasetikai Bizottsag | Pecsi Tudomanyegyetem | Budapest |  | 1051 | Hungary |
| 3804 | Egeszsegugyi Tudomanyos Tanacs Tudomanyos es Kutatasetikai Bizottsag | Szegedi Tudomanyegyetem Szent-Gyorgyi Albert Klinikai Kozpont | Budapest |  | 1051 | Hungary |

| **Center Number** | **Ethics Committee or Institutional Review Board** | **Department / Organization** | **EC/IRB**  **City** | **EC/IRB**  **State/ Province** | **EC/IRB**  **Postal Code** | **Center Country** |
| --- | --- | --- | --- | --- | --- | --- |
| 3900 | Beacon Hospital Research Ethics Committee | Beacon Clinic | Sandyford |  | 18 | Ireland |
| 3901 | Research Ethics Committee Southeast Area | Whitfield Clinic | Waterford |  | X91 | Ireland |
| 3902 | Mater Misericordiae Univ Hospital Research Ethics Committee | Mater Private Hospital | Dublin | Dublin | 7 | Ireland |
| 3903 | Research Ethics Committee Southeast Area | Waterford Regional Hospital | Waterford |  | X91 | Ireland |
| 8400 | Helsinki Committee, Rabin MC | Rabin Medical Center-Beilinson Campus | Petah Tikva |  | 49100 | Israel |
| 8401 | Helsinki Committee, Sourasky MC | Tel Aviv Sourasky Medical Center | Tel-Aviv |  | 64239 | Israel |
| 8402 | Helsinki Committee, Bnai- Zion | Bnai Zion Medical Center | Haifa |  | 31048 | Israel |
| 6003 | Prasad Eye Institute Ethics Committee | L. V. Prasad Eye Institute | Hyderabad | Andhra Pradesh | 500034 | India |
| 6002 | All India Institute of Medical Sciences Ethics Committee | All India Institute of Medical Sciences | Ansari Nagar | Delhi | 110029 | India |
| 6000 | Aravind Eye Care System Ethical Committee | Aravind Eye Hospital | Coimbator e | Tamilnadu | 641014 | India |
| 6004 | Sangini Hospital Ethics Committee | Bankers Eye Institute | Ahmedaba d | Gujarat | 380006 | India |
| 6005 | Sparsh Hospital Ethics Committee | L. V. Prasad Eye Institute | Bhubanes war | Orissa | 751007 | India |
| 6008 | Vision Research Foundation Ethics Sub- Committee | Sankara Nethralaya | Chennai | Tamilnadu | 600006 | India |
| 6010 | National Institute of Ophthalmology Ethics Committee | National Institute of Ophthalmology | Pune | Maharasht ra | 411005 | India |
| 4001 | Comitato Etico Interaziendale | Reparto diOculistica dell Ospedale SS Antonio e Biagio e Cesare Arrigo de Alessandria | Alessandri a |  | 15121 | Italy |
| 4002 | Comitato Etico Indipendente - Azienda Ospedaliero-Univeritaria | Ente Ecclesiastico Ospedale Generale Regionale F Miulli | Bari | Bari | 70124 | Italy |
| 4003 | Reggio Calabria Comitato Etico | Azienda Ospedaliera Bianchi Melacrino Morelli | Reggio Calabria |  | 89100 | Italy |
| 4004 | Comitato Etico Dell 'Universita' Cattolica Del Sacro Cuore | Policlinico Universitario Agostino Gemelli | Roma |  | 001 68 | Italy |
| 4005 | Spedali Civili - Brescia Comitato Etico Provinciale | Azienda Socio Sanitaria Territoriale degli Spedali Civili di Brescia (Presidio Spedali Civili) | Brescia |  | 25123 | Italy |
| 4006 | Comitato per la Sperimentazione Clinica Medicinali dell'Az Ospedaliero Universitaria Pisana d. Pisa | Azienda Ospedaliero Universitaria Cisanello | Pisa |  | 56126 | Italy |
| 4008 | Regione Autonoma Della Sardegna Azienda Ospedaliero Universitaria Di Cagliari Comitato Etico Indipendente | Ospedale S.Giovanni di Dio | Cagliari |  | 091 24 | Italy |
| 4009 | Comitato Etico Dell 'Universita' Dell' Universita' "Sapienza" | Umberto I Pol. di Roma-Universita di Roma La Sapienza | Roma |  | 001 61 | Italy |
| 4010 | Regione del Veneto Aziendo U.L.S.S. n. 1 | Ospedale San Martino di Belluno | Belluno |  | 27717 | Italy |

| **Center Number** | **Ethics Committee or Institutional Review Board** | **Department / Organization** | **EC/IRB**  **City** | **EC/IRB**  **State/ Province** | **EC/IRB**  **Postal Code** | **Center Country** |
| --- | --- | --- | --- | --- | --- | --- |
| 6154 | Fukushima Medical University Hospital Institutional Review Board | Fukushima Medical University Hospital | Fukushima  -shi | Fukushim a-Ken | 960-1295 | Japan |
| 6155 | Sapporo City General Hospital The Ethical Committee of Sapporo City General Hospital | Sapporo City General Hospital | Sapporo- shi | Hokkaido | 060-8604 | Japan |
| 6115 | Asahikawa Medical University Hospital Institutional Review Board | Asahikawa Medical University Hospital | Asahikawa  -shi | Hokkaido | 078-8510 | Japan |
| 6161 | Tohoku University Hospital Institutional Review Board | Tohoku University Hospital | Sendai-shi | Miyagi- Ken | 980-8574 | Japan |
| 6116 | Akita University Hospital Institutional Review Board | Akita University Hospital | Akita-shi | Akita-Ken | 010-8543 | Japan |
| 6100 | Hattori Clinic Institutional Review Board | Shuhokai Ohtsuka Eye Hospital | Sapporo- shi | Hokkaido | 001-0016 | Japan |
| 6175 | Aomori Prefectural Central Hospital the ethical committee in Aomori Prefectural Central Hospital | Aomori Prefectural Central Hospital | Aomori | Aomori | 030-8553 | Japan |
| 6117 | Kitami Red Cross Hospital Institutional Review Board | Kitami Red Cross Hospital | Kitami-shi | Hokkaido | 090-8666 | Japan |
| 6118 | Jusendo General Hospital Independent Ethics Committee | Jusendo General Hospital | Koriyama- shi | Fukushim a-Ken | 963-8585 | Japan |
| 6182 | Yamagata University Hospital Institutional Review Board | Yamagata University Hospital | Yamagata- shi | Yamagata  -Ken | 990-9585 | Japan |
| 6101 | Hattori Clinic Institutional Review Board | Yoshida Eye Hospital | Hakodate- shi | Hokkaido | 041-0851 | Japan |
| 6119 | Gunma University Hospital Institutional Review Board | Gunma University Hospital | Maebashi- shi | Gunma- Ken | 371-8511 | Japan |
| 6107 | Tokyo Medical University Ibaraki Medical Center Institutional Review Board | Tokyo Medical University Ibaraki Medical Center | Inashiki- gun | Ibaraki- Ken | 300-0395 | Japan |
| 6102 | Hattori Clinic Institutional Review Board | Aoyagi Eye Clinic | Ueda-shi | Nagano- Ken | 386-0002 | Japan |
| 6120 | Dokkyo Medical University Hospital Institutional Review Board | Dokkyo Medical University Hospital | Shimotsug a-gun | Tochigi- Ken | 321-0293 | Japan |
| 6121 | Jichi Medical University Hospital Institutional Review Board | Jichi Medical University Hospital | Shimotsuk e-shi | Tochigi- Ken | 329-0498 | Japan |
| 6103 | Matsumoto Dental University Hospital Institutional Review Board | Matsumoto Dental University Hospital | Shiojiri-shi | Nagano- Ken | 399-0781 | Japan |
| 6122 | Tsukuba University Hospital Institutional Review Board | Tsukuba University Hospital | Tsukuba- shi | Ibaraki- Ken | 305-8576 | Japan |
| 6162 | Saitama Medical University Hospital Institutional Review Board | Saitama Medical University Hospital | Iruma-gun | Saitama- Ken | 350-0495 | Japan |
| 6163 | Toho University Sakura Medical Center Independent Ethics Committee | Toho University Sakura Medical Center | Sakura-shi | Chiba-Ken | 285-8741 | Japan |
| 6199 | Juntendo University Urayasu Hospital Institutional Review Board | Juntendo University Urayasu Hospital | Urayasu- shi | Chiba-Ken | 279-0021 | Japan |
| 6123 | Saitama Red Cross Hospital Institutional Review Board | Saitama Red Cross Hospital | Saitama- shi | Saitama- Ken | 338-8553 | Japan |
| 6156 | Hattori Clinic Institutional Review Board | Hoeikai Hoshiai Eye Clinic | Saitama- shi | Saitama- Ken | 336-0963 | Japan |
| 6104 | Yokohama City University Medical Center Institutional Review Board | Yokohama City University Medical Center | Yokohama  -shi | Kanagawa  -Ken | 232-0024 | Japan |

| **Center Number** | **Ethics Committee or Institutional Review Board** | **Department / Organization** | **EC/IRB**  **City** | **EC/IRB**  **State/ Province** | **EC/IRB**  **Postal Code** | **Center Country** |
| --- | --- | --- | --- | --- | --- | --- |
| 6124 | Yokohama City University Hospital Institutional Review Board | Yokohama City University Hospital | Yokohama  -shi | Kanagawa  -Ken | 236-0004 | Japan |
| 6125 | Yokosuka Kyosai Hospital Institutional Review Board | Yokosuka Kyosai Hospital | Yokohama  -shi | Kanagawa  -Ken | 236-0004 | Japan |
| 6183 | Seirei Hamamatsu General Hospital Institutional Review Board | Seirei Hamamatsu General Hospital | Hamamats u-shi | Shizuoka- Ken | 430-8558 | Japan |
| 6108 | St. Marianna University School of Medicine Hospital Institutional Review Board | St. Marianna University School of Medicine Hospital | Kawasaki- shi | Kanagawa  -Ken | 216-8511 | Japan |
| 6126 | Shizuoka Saiseikai General Hospital Internal Review Board | Shizuoka Saiseikai General Hospital | Shizuoka- shi | Shizuoka- Ken | 422-8527 | Japan |
| 6127 | Juntendo University Shizuoka Hospital Institutional Review Board | Juntendo University Shizuoka Hospital | Izunokuni- shi | Shizuoka- Ken | 410-2295 | Japan |
| 6109 | Hattori Clinic Institutional Review Board | Ishikawa Eye Clinic | Shizuoka- shi | Shizuoka- Ken | 420-0841 | Japan |
| 6176 | Hattori Clinic Institutional Review Board | Kaiya Ophthalmology | Hamamats u-shi | Shizuoka- Ken | 430-0903 | Japan |
| 6105 | Hattori Clinic Institutional Review Board | Totsukaekimae Suzuki Eye Clinic | Yokohama  -shi | Kanagawa  -Ken | 244-0003 | Japan |
| 6177 | Nihon University Hospital Institutional Review Board | Nihon University Hospital | Chiyoda- ku | Tokyo-To | 101-8309 | Japan |
| 6164 | Kyorin University Hospital Institutional Review Board | Kyorin University Hospital | Mitaka-shi | Tokyo-To | 181-8611 | Japan |
| 6165 | Tokyo Medical University Hospital Institutional Review Board | Tokyo Medical University Hospital | Shinjuku- ku | Tokyo-To | 160-0023 | Japan |
| 6128 | University of Yamanashi Hospital Institutional Review Board | University of Yamanashi Hospital | Chuo-shi | Yamanash i-Ken | 409-3898 | Japan |
| 6129 | Saiando Ochanomizu Inoue Eye Clinic Institutional Review Board | Saiando Ochanomizu Inoue Eye Clinic | Chiyoda- ku | Tokyo-To | 101-0062 | Japan |
| 6130 | Tokyo Medical University Hachioji Medical Center Institutional Review Board | Tokyo Medical University Hachioji Medical Center | Hachioji- shi | Tokyo-To | 193-0998 | Japan |
| 6187 | Juntendo University Hospital Institutional Review Board | Juntendo University Hospital | Bunkyo-ku | Tokyo-To | 113-8431 | Japan |
| 6173 | Tokyo Women's Medical University Hospital Institutional Review Board | Tokyo Women's Medical University Hospital | Shinjuku- ku | Tokyo-To | 162-8666 | Japan |
| 6131 | Nagoya University Hospital Institutional Review Board | Nagoya University Hospital | Nagoya- shi | Aichi-Ken | 466-8560 | Japan |
| 6132 | Mie University Hospital Internal Review Board | Mie University Hospital | Tsu-shi | Mie-Ken | 514-8507 | Japan |
| 6133 | Hattori Clinic Institutional Review Board | Shozankai Miyake Eye Hospital | Nagoya- shi | Aichi-Ken | 462-0825 | Japan |
| 6134 | JCHO Chukyo Hospital Institutional Review Board | JCHO Chukyo Hospital | Nagoya- shi | Aichi-Ken | 457-8510 | Japan |
| 6188 | Fujita Health University Hospital Institutional Review Board | Fujita Health University Hospital | Toyoake- shi | Aichi-Ken | 470-1192 | Japan |
| 6110 | Hattori Clinic Institutional Review Board | Koyokai Yayoi Hospital | Toyohashi  -shi | Aichi-Ken | 441-8106 | Japan |
| 6178 | Aichi Medical University Hospital Institutional Review Board | Aichi Medical University Hospital | Nagakute- shi | Aichi-Ken | 480-1195 | Japan |
| 6136 | Hattori Clinic Institutional Review Board | Nishijima Eye Clinic | Kyoto-shi | Kyoto-Fu | 604-0837 | Japan |
| 6157 | Shiga University of Medical Science Hospital Institutional Review Board | Shiga University of Medical Science Hospital | Otsu-shi | Shiga-Ken | 520-2192 | Japan |

| **Center Number** | **Ethics Committee or Institutional Review Board** | **Department / Organization** | **EC/IRB**  **City** | **EC/IRB**  **State/ Province** | **EC/IRB**  **Postal Code** | **Center Country** |
| --- | --- | --- | --- | --- | --- | --- |
| 6189 | Kyoto University Hospital Institutional Review Board | Kyoto University Hospital | Kyoto-shi | Kyoto-Fu | 606-8507 | Japan |
| 6158 | Kanazawa University Hospital Institutional Review Board | Kanazawa University Hospital | Kanazawa  -shi | Ishikawa- Ken | 920-8641 | Japan |
| 6190 | Toyama University Hospital Institutional Review Board | Toyama University Hospital | Toyama- shi | Toyama- Ken | 930-0194 | Japan |
| 6166 | Osaka University Hospital Institutional Review Board | Osaka University Hospital | Suita-shi | Osaka-Fu | 565-0871 | Japan |
| 6179 | Kansai Medical University Hospital Institutional Review Board | Kansai Medical University Hospital | Hirakata- shi | Osaka-Fu | 573-1191 | Japan |
| 6191 | Hyogo College of Medicine Hospital Institutional Review Board | Hyogo College of Medicine Hospital | Nishinomiy a-shi | Hyogo- Ken | 663-8501 | Japan |
| 6200 | Hyogo Prefectural Amagasaki General Medical Center Institutional Review Board | Hyogo Prefectural Amagasaki General Medical Center | Amagasak i-shi | Hyogo- Ken | 660-8550 | Japan |
| 6137 | Kansai Medical University Medical Center Institutional Review Board | Kansai Medical University Takii Hospital | Moriguchi- shi | Osaka-Fu | 570-8507 | Japan |
| 6138 | Steel Memorial Hirohata Hospital Institutional Review Board | Steel Memorial Hirohata Hospital | Himeji-shi | Hyogo- Ken | 671-1122 | Japan |
| 6159 | Nara Medical University Hospital Institutional Review Board | Nara Medical University Hospital | Kashihara- shi | Nara-Ken | 634-8522 | Japan |
| 6184 | Kobe Kaisei Hospital Institutional Review Board | Kobe Kaisei Hospital | Kobe-shi | Hyogo- Ken | 657-0068 | Japan |
| 6160 | Kindai University Hospital Independent Ethics Committee | Kindai University Hospital | Osakasay ama-shi | Osaka-Fu | 589-8511 | Japan |
| 6202 | Japan Red Cross Society Wakayama Medical Center Independent Ethics Committee | Japan Red Cross Society Wakayama Medical Center | Wakayam a-shi | Wakayam a-Ken | 640-8558 | Japan |
| 6180 | Kakogawa City West Hospital Institutional Review Board | Kakogawa City West Hospital | Kakogawa  -shi | Hyogo- Ken | 675-8611 | Japan |
| 6139 | Kagawa University Hospital Institutional Review Board | Kagawa University Hospital | Kita-gun | Kagawa- Ken | 761-0793 | Japan |
| 6140 | Hiroshima University Hospital Institutional Review Board | Hiroshima University Hospital | Hiroshima- shi | Hiroshima- Ken | 734-8551 | Japan |
| 6141 | Ehime University Hospital Independent Ethics Committee | Ehime University Hospital | Toon-shi | Ehime- Ken | 791-0295 | Japan |
| 6185 | Tokushima University Hospital Institutional Review Board | Tokushima University Hospital | Tokushima  -shi | Tokushim a-Ken | 770-8503 | Japan |
| 6167 | Kochi Medical School Hospital Institutional Review Board | Kochi Medical School Hospital | Nankoku- shi | Kochi-Ken | 783-8505 | Japan |
| 6111 | Matsuyama Red Cross Hospital Institutional Review Board | Matsuyama Red Cross Hospital | Matsuyam a-shi | Ehime- Ken | 790-8524 | Japan |
| 6192 | Kyushu University Hospital Institutional Review Board | Kyushu University Hospital | Higashi-ku | Fukuoka | 812-8582 | Japan |
| 6143 | Japanese Red Cross Nagasaki Genbaku Hospital Independent Ethics Committee | Japanese Red Cross Nagasaki Genbaku Hospital | Nagasaki- shi | Nagasaki- Ken | 852-8511 | Japan |
| 6144 | Kagoshima University Hospital Institutional Review Board | Kagoshima University Medical And Dental Hospital | Kagoshim a-shi | Kagoshim a-Ken | 890-8520 | Japan |
| 6193 | University of Miyazaki Hospital Institutional Review Board | University of Miyazaki Hospital | Miyazaki- shi | Miyazaki- Ken | 889-1692 | Japan |

| **Center Number** | **Ethics Committee or Institutional Review Board** | **Department / Organization** | **EC/IRB**  **City** | **EC/IRB**  **State/ Province** | **EC/IRB**  **Postal Code** | **Center Country** |
| --- | --- | --- | --- | --- | --- | --- |
| 6168 | Meiwakai Miyata Ophthalmic Hospital Institutional Review Board | Meiwakai Miyata Ophthalmic Hospital | Miyakonoj o-shi | Miyazaki- Ken | 885-0051 | Japan |
| 6194 | Fukuoka University Hospital Institutional Review Board | Fukuoka University Hospital | Fukuoka- shi | Fukuoka- Ken | 814-0180 | Japan |
| 6145 | Nagasaki University Hospital Institutional Review Board | Nagasaki University Hospital | Nagasaki- shi | Nagasaki- Ken | 852-8501 | Japan |
| 6195 | University of Occupational and Environmental Health Hospital Internal Review Board | University of Occupational and Environmental Health Hospital | Kitakyushu  -shi | Fukuoka- Ken | 807-8556 | Japan |
| 6169 | Meiwakai Kagoshima Miyata Ophthalmic Hospital Institutional Review Board | Meiwakai Kagoshima Miyata Ophthalmic Hospital | Kagoshim a-shi | Kagoshim a-Ken | 890-0046 | Japan |
| 6106 | Hattori Clinic Institutional Review Board | Taidokai Sato Ganka Iin Domachi Clinic | Yamagata- shi | Yamagata  -Ken | 990-0051 | Japan |
| 6146 | Yonezawa City Hospital Institutional Review Board | Yonezawa City Hospital | Yonezawa  -shi | Yamagata  -Ken | 992-8502 | Japan |
| 6170 | JOHAS Tohoku Rosai Hospital Institutional Review Board | JOHAS Tohoku Rosai Hospital | Sendai-shi | Miyagi- Ken | 981-8563 | Japan |
| 6114 | Meiji University of Integrative Medicine Institutional Review Board | Meiji University of Integrative Medicine | Nantan-shi | Kyoto-Fu | 629-0392 | Japan |
| 6174 | Obihiro Kyokai Hospital Institutional Review Board | Obihiro Kyokai Hospital | Obihiro-shi | Hokkaido | 080-0805 | Japan |
| 6171 | Sapporo Medical University Hospital Institutional Review Board | Sapporo Medical University Hospital | Sapporo- shi | Hokkaido | 060-8543 | Japan |
| 6148 | Ogaki Tokushukai Hospital Tokushu-kai Ethics Committee | Ogaki Tokushukai Hospital | Ogaki-shi | Gifu-Ken | 503-0015 | Japan |
| 6112 | Hattori Clinic Institutional Review Board | Tagawa Eye Clinic | Kanazawa  -shi | Ishikawa- Ken | 920-1151 | Japan |
| 6186 | University of Fukui Hospital Institutional Review Board | University of Fukui Hospital | Yoshida- gun | Fukui-Ken | 910-1193 | Japan |
| 6150 | Japanese Red Cross Society Suwa Hospital Institutional Review Board | Japanese Red Cross Society Suwa Hospital | Suwa-shi | Nagano- Ken | 392-8510 | Japan |
| 6151 | Hattori Clinic Institutional Review Board | Hirota Eye Clinic | Shunan- shi | Yamaguch i-Ken | 745-0017 | Japan |
| 6113 | Hattori Clinic Institutional Review Board | Dannoue Eye Clinic | Kawasaki- shi | Kanagawa  -Ken | 211-0053 | Japan |
| 6152 | Hattori Clinic Institutional Review Board | Jigankai Sanjo Eye Clinic | Sanjo-shi | Niigata- Ken | 955-0852 | Japan |
| 6153 | Hattori Clinic Institutional Review Board | Infinity Medical Group Kondo Eye Clinic | Hachioji- shi | Tokyo-To | 192-0081 | Japan |
| 6172 | Hattori Clinic Institutional Review Board | Musashi Dream Eye Clinic | Osaka-shi | Osaka-Fu | 543-0027 | Japan |
| 6181 | Toho University Ohashi Medical Center Institutional Review Board | Toho University Ohashi Medical Center | Meguro-ku | Tokyo-To | 153-8515 | Japan |
| 6196 | Hattori Clinic Institutional Review Board | Shinseikai Toyama Hospital | Imizu-shi | Toyama- Ken | 939-0243 | Japan |
| 6197 | Hattori Clinic Institutional Review Board | Ando Eye Clinic | Ashigarak ami-gun | Kanagawa  -Ken | 258-0003 | Japan |
| 6198 | Juntendo University Nerima Hospital Institutional Review Board | Juntendo University Nerima Hospital | Nerima-ku | Tokyo-To | 177-8521 | Japan |
| 6600 | Kangnam Sacred Heart Hospital IRB Membership List | Hallym University Kangnam Sacred Heart Hospital | Seoul |  | 150-950 | Korea, Republic of |
| 6601 | Seoul National University Bundang Hospital | Seoul National University Bundang Hospital | Seongnam  -si | Gyeonggi- do | 13620 | Korea, Republic of |

| **Center Number** | **Ethics Committee or Institutional Review Board** | **Department / Organization** | **EC/IRB**  **City** | **EC/IRB**  **State/ Province** | **EC/IRB**  **Postal Code** | **Center Country** |
| --- | --- | --- | --- | --- | --- | --- |
| 6602 | Seoul National University Hospital Institutional Review Board | Seoul National University Hospital | Seoul | Gyeonggi- do | 110744 | Korea, Republic of |
| 6603 | Kim's Eye Hospital IRB | Kim's Eye Hospital | Seoul |  | 150-034 | Korea, Republic of |
| 6604 | Seoul St. Mary's Hospital, The Catholic University of Korea IRB | The Catholic University of Korea, Seoul St. Mary’s Hospital | Seocho-gu |  | 137-701 | Korea, Republic of |
| 6605 | The Institutional Review Board of Kyungpook National University Hospital | Kyungpook National University Hospital | Daegu | Gyeongsa ngbuk-do | 700-721 | Korea, Republic of |
| 6606 | Yonsei University Gangnam Severance Hospital, Institutional Review Board | Gangnam Severance Hospital, Yonsei University Health System | Gangnam- gu |  | 062 73 | Korea, Republic of |
| 6607 | PNUH Institutional Review Board | Pusan National University Hospital | Busan |  | 602-739 | Korea, Republic of |
| 6608 | IRB of Inje University Busan Paik Hospital | Inje University Busan Paik Hospital | Busan |  | 47392 | Korea, Republic of |
| 6609 | Yeungnam University Hospital Institutional Review Board | Yeungnam University Hospital | Daegu |  | 42415 | Korea, Republic of |
| 6610 | Asan Medical Center IRB | Asan Medical Center | Seoul |  | 055 05 | Korea, Republic of |
| 6612 | KHUH IRB | Kyung Hee University Hospital | Seoul |  | 024 47 | Korea, Republic of |
| 6652 | Samsung Medical Center Institutional Review Board | Samsung Medical Center | Seoul |  | 063 51 | Korea, Republic of |
| 6611 | The Institutional Review Board of Ajou University Hospital | Ajou University Hospital | Gyeonggi- do | Gyeonggi- do | 16499 | Korea, Republic of |
| 1400 | Comite de Etica en Investigacion de la facultad de Medicina y Hospital Universitario de la Universidad Autonoma de Nuevo Leon | Hospital Universitario Dr Jose E Gonzalez | Monterrey | Nuevo León | 64460 | Mexico |
| 1401 | Comite de Etica en Investigacion de la Escuela de Medicina del Instituto Tecnologico y de Estudios Superiores de Monterrey | CIIES | Monterrey | Nuevo León | 64710 | Mexico |
| 1403 | Comite de Investigacion Instituo de Oftalmologia Fundacion de Asistencia Privada | Instituto de Oftalmología Fundación Conde de la Valenciana | Mexico City | Distrito Federal | 0 6800 | Mexico |
| 1405 | Comite de Etica e Investigacion Fundacion Hospital "Nuestra Senora de la Luz" | Hospital Oftalmológico Nuestra Señora de la Luz | Mexico City | Distrito Federal | 0 6030 | Mexico |
| 1406 | Comite de Investigaction de la Clinica Bajio CLINBA S.C. | Clínica de Ojos Monterrey S.A. de C.V. | Guanajuat o | Nuevo León | 36090 | Mexico |
| 1412 | Comite Independiente de Etica de Investigación y Bioseguridad del Bajio SC | Dr. Alejandro Dalma y asoc. | Guanajuat o | Distrito Federal | 36090 | Mexico |
| 1413 | Comite Independiente de Etica de Investigación y Bioseguridad del Bajio SC | RetimediQ Centro de Retina y Oftalmologia Especializada | Guanajuat o | Yucatán | 36090 | Mexico |
| 5100 | Medical Ethics Committee, University Malaya Medical Centre | University of Malaya Eye Research Centre | Kuala Lumpur | Kuala Lumpur | 59100 | Malaysia |
| 5101 | Medical Research Ethics Committee, Ministry of Health Malaysia | Hospital Selayang | Kuala Lumpur | Selangor | 59000 | Malaysia |

| **Center Number** | **Ethics Committee or Institutional Review Board** | **Department / Organization** | **EC/IRB**  **City** | **EC/IRB**  **State/ Province** | **EC/IRB**  **Postal Code** | **Center Country** |
| --- | --- | --- | --- | --- | --- | --- |
| 5102 | Medical Research & Ethics Committee Kementerian Kesihatan Malaysia | International Specialist Eye Centre | Kuala Lumpur | Kuala Lumpur | 59000 | Malaysia |
| 5103 | Universiti Kebangsaan Malaysia Medical Centre | Pusat Perubatan Universiti Kebangsaan Malaysia | Kuala Lumpur | Kuala Lumpur | 56000 | Malaysia |
| 4100 | Medisch Ethische Toetsingscommissie | Sint Elisabeth Ziekenhuis Afd. Oogheelkunde | Tilburg | Noord- Brabant | 5022 GC | Netherlands |
| 4101 | Medisch Ethische Toetsingscommissie | OMC Amsterdam | Tilburg | Noord- Brabant | 5022 GC | Netherlands |
| 4103 | Medisch Ethische Toetsingscommissie | Flevoziekenhuis | Tilburg | Noord- Brabant | 5022 GC | Netherlands |
| 8500 | Hospital Nacional Guillermo Almenara Irigoyen Comite de Etica en Investigacion | Hospital Nacional Guillermo Almenara Irigoyen | Lima |  | 15036 | Peru |
| 8501 | Comite Institucional de Etica en Investigacion de la Universidad de San Martin de Porres | Instituto Oftalmosalud S.R.L | Lima |  | 15036 | Peru |
| 8502 | Comite Institucional de Etica en Investigacion de la Universidad de San Martin de Porres | Macula D&T | Lima |  | 15036 | Peru |
| 8503 | Prisma ONG | Ophtalmology-TG Laser Oftalmica | Lima |  | 15036 | Peru |
| 4200 | Ethics Committee of Silesian Medical Chamber | Samodzielny Publiczny ZOZ | Grazynski ego |  | 40-126 | Poland |
| 4205 | Ethics Committee of Silesian Medical Chamber | NZOZ Lens-Med | Grazynski ego |  | 40-126 | Poland |
| 4201 | Ethics Committee of Silesian Medical Chamber | 10 Wojskowy Szpital Kliniczny | Grazynski ego |  | 40-126 | Poland |
| 4206 | Ethics Committee of Silesian Medical Chamber | Szpital Specjalistyczny im Sokołowskiego | Grazynski ego |  | 40-126 | Poland |
| 4202 | Ethics Committee of Silesian Medical Chamber | NZOZ Ocu Service | Grazynski ego |  | 40-126 | Poland |
| 4203 | Ethics Committee of Silesian Medical Chamber | Specjalistyczny Cabinet Lekarski Krystyna Raczynska | Grazynski ego |  | 40-126 | Poland |
| 4204 | Ethics Committee of Silesian Medical Chamber | NZOZ Medilens | Grazynski ego |  | 40-126 | Poland |
| 4211 | Ethics Committee of Silesian Medical Chamber | Wojewódzki Szpital Okulistyczny w Krakowie | Grazynski ego |  | 40-126 | Poland |
| 4212 | Ethics Committee of Silesian Medical Chamber | Centrum Diagnostyki i Mikrochirurgii Oka LENS | Grazynski ego |  | 40-126 | Poland |
| 4213 | Ethics Committee of Silesian Medical Chamber | Szpital Specjalistyczny IM  J.K. Lukowicza | Grazynski ego |  | 40-126 | Poland |
| 4217 | Ethics Committee of Silesian Medical Chamber | Centrum Medyczne Uno-Med (Private Practice) | Grazynski ego |  | 40-126 | Poland |
| 4219 | Ethics Committee of Silesian Medical Chamber | Szpital Zakonu Bonifratrów im. Św. Jana Bożego w Łodzi | Grazynski ego |  | 40-126 | Poland |
| 4220 | Ethics Committee of Silesian Medical Chamber | Wojskowy Instytut Medyczny | Grazynski ego |  | 40-126 | Poland |
| 4221 | Ethics Committee of Silesian Medical Chamber | Medical University of Lublin | Grazynski ego |  | 40-126 | Poland |
| 4300 | Aibili Comissao de Etica para a Saude | AIBILI | Porto Salvo |  | 2740-262 | Portugal |
| 4303 | Hospital Lusiadas Lisboa Comissao de Etica para a  Saude | HPP - Hospital dos Lusíadas | Lisbon |  | 1500-458 | Portugal |

| **Center Number** | **Ethics Committee or Institutional Review Board** | **Department / Organization** | **EC/IRB**  **City** | **EC/IRB**  **State/ Province** | **EC/IRB**  **Postal Code** | **Center Country** |
| --- | --- | --- | --- | --- | --- | --- |
| 4304 | Centro Hospitalar de Entre o Douro e Vouga, E.P.E. | Centro Hospitalar de Entre o Douro e Vouga, E.P.E - Hospital de São Sebastião | Santa Maria da Feira |  | 4520-211 | Portugal |
| 4306 | Centro Hospitalar Leiria Comissao de Etica | Centro Hospitalar Leiria - Hospital Santo André | Leira |  | 2410-197 | Portugal |
| 4301 | Centro Hospitalar Leiria Comissao de Etica | Hospital Pedro Hispano | Leira |  | 2410-197 | Portugal |
| 4302 | Centro Hospitalar Do Baixo Vouga, E.P.E./Aveiro | Centro Hospitalar do Baixo Vouga, E.P.E.  – Unidade de Aveiro | Aveiro |  | 3814-501 | Portugal |
| 4305 | CEIC - Parque de Saude de Lisboa | Espaço Médico de Coimbra | Lisboa |  | 1749-004 | Portugal |
| 4407 | The Committee of Biomedical Ethics of Ufa Institute of scientific- research of eye diseases of Academy of Sciences (Bashkortostan) | SBI "Ufa scientific research institute of eye diseases of academy of sciences of the republic of Bas | Ufa |  | 450077 | Russia |
| 4400 | The Ethics Committee of "The Postgraduating Doctors' Training Institute" of the Healthcare and Social Development Ministry of the Chuvash Republic | The S.N.Fyodorov Federal State Institution Eye Microsurgery Complex (Cheboxary) | Cheboksar y |  | 428003 | Russia |
| 4412 | The Ethics Committee of GBUZ SOCOB n.a. T.I.  Eroshevsky | SBEI HPE "Samara State Medical University" of the MoH of the RF |  |  | 443099 | Russia |
| 4423 | The Independent Multidisciplinary Committee on Ethical Review of Clinical Trials | The Irkutsk Affiliate of Federal State Budgetary Institution "MNTK ye  Microsurgery Complex" n.a. S.N | Moscow |  | 125468 | Russia |
| 4403 | The Independent Multidisciplinary Committee on Ethical Review of Clinical Trials | Institution of Republic Sakha (Yakutiya) Yakutsk Republican Ophthalmology Hospital | Moscow |  | 125468 | Russia |
| 4402 | The Independent Multidisciplinary Committee on Ethical Review of Clinical Trials | The S.N.Fyodorov Federal State Institution Eye Microsurgery Complex (Khabarovsk) | Moscow |  | 125468 | Russia |
| 4404 | The Independent Multidisciplinary Committee on Ethical Review of Clinical Trials | Territorial Diabetic Center | Moscow |  | 125468 | Russia |
| 4401 | The Independent Multidisciplinary Committee on Ethical Review of Clinical Trials | Dignostic Center №7 | Moscow |  | 125468 | Russia |
| 4406 | The Ethics Committee of "Federal State budgetary Institution "Scientific Research Institute of Eye Diseases" of Russian Academy of medical Sciences | Scientific Research Institute of Eye Diseases | Moscow |  | 119021 | Russia |
| 4408 | The Ethics Committee of The Helmholtz Moscow Research Institute of Eye Diseases | Moscow Helmholtz Research Institute of Ophthalmology | Moscow |  | 105062 | Russia |

| **Center Number** | **Ethics Committee or Institutional Review Board** | **Department / Organization** | **EC/IRB**  **City** | **EC/IRB**  **State/ Province** | **EC/IRB**  **Postal Code** | **Center Country** |
| --- | --- | --- | --- | --- | --- | --- |
| 4405 | The Independent Multidisciplinary Committee on Ethical Review of Clinical Trials | Tyumen Regional Ophthalmology Dispensary | Moscow |  | 125468 | Russia |
| 4419 | The Independent Multidisciplinary Committee on Ethical Review of Clinical Trials | Chita State Medical Academy | Moscow |  | 125468 | Russia |
| 4418 | The Independent Multidisciplinary Committee on Ethical Review of Clinical Trials | The S.N.Fyodorov Federal State Institution Eye Microsurgery Complex (Tambov) | Moscow |  | 125468 | Russia |
| 4420 | The Independent Multidisciplinary Committee on Ethical Review of Clinical Trials | SBHI "Penza Regional Ophtalmological Hospital" | Moscow |  | 125468 | Russia |
| 4421 | The Independent Multidisciplinary Committee on Ethical Review of Clinical Trials | BI of Khanty- Mansyisk region Yugra "Surgut regional clinical hospital" | Moscow |  | 125468 | Russia |
| 4422 | The Independent Multidisciplinary Committee on Ethical Review of Clinical Trials | Krasnodar Branch of The S.N. Fyodorov FSBI "Eye  microsurgery complex" | Moscow |  | 125468 | Russia |
| 4425 | The Independent Multidisciplinary Committee on Ethical Review of Clinical Trials | SBEI HPE "Saratov State Medical University n.a. V. I. Razumovskiy" of the MoH of the RF | Moscow |  | 125468 | Russia |
| 4426 | State Autonomous Healthcare Institution "Republican Clinical Opthalmologic Hospital of the Ministry of Health of Tatarstan Republic" | SAIH "Republican clinical ophthalmological hospital of MoH of Rebublic of Tatarstan" | Kazan |  | 420012 | Russia |
| 4424 | Biomedical Ethics Committee of Federal State Budgetary Institution of scientific and technical complex of Microsurgical eye | MBHI City Clinical Hospital #11 | Novosibirs k |  | 630071 | Russia |
| 4427 | Biomedical Ethics Committee of Federal State Budgetary Institution of scientific and technical complex of Microsurgical eye | The S.N.Fyodorov Federal State Institution Eye Microsurgery Complex (Novosibirsk) | Novosibirs k |  | 630071 | Russia |
| 8901 | Prince Sultan Military Medical City Research Ethics Committee | Prince Sultan Military Medical City | Riyadh |  | 11159 | Saudi Arabia |
| 8902 | Kingdom of Saudi Arabia  - Ministry of National Guard - Health Affairs | King Abdullah International Medical Research Center IRB Office | Riyadh |  | 22490 | Saudi Arabia |
| 8900 | King Khaled Eye Specialist Hospital HEC/IRB | King Khaled Eyes Specialist Hospital | Riyadh |  | 11462 | Saudi Arabia |
| 8903 | Kingdom of Saudi Arabia  - Ministry of National Guard - Health Affairs | King Abdullah International Medical Research Center IRB Office | Riyadh |  | 22490 | Saudi Arabia |
| 6300 | Parkway Hospitals Singapore Pte Ltd | PIEC | Singapore |  | 238164 | Singapore |
| 6301 | National Healthcare Group | NUHS | Singapore |  | 149547 | Singapore |
| 6302 | SingHealth Centralised Institutional Review Board | Singapore National Eye Centre | Singapore |  | 168753 | Singapore |
| 8600 | Republic of Slovenia The National Medical Ethics Committee | Univerzitetni Klinicni Center Ljubljana Ocesna Klinika | Ljubljana |  | SI-1525 | Slovenia |

| **Center Number** | **Ethics Committee or Institutional Review Board** | **Department / Organization** | **EC/IRB**  **City** | **EC/IRB**  **State/ Province** | **EC/IRB**  **Postal Code** | **Center Country** |
| --- | --- | --- | --- | --- | --- | --- |
| 8601 | Republic of Slovenia The National Medical Ethics Committee | University Medical Centre Maribor | Ljubljana |  | SI-1525 | Slovenia |
| 8602 | Ethical Committee General Hospital Celje | General Hospital Celje | Celje |  | 3000 | Slovenia |
| 8603 | Republic of Slovenia The National Medical Ethics Committee | General Hospital Novo Mesto | Ljubljana |  | SI-1525 | Slovenia |
| 4500 | *Nemocnica Ruzinov* | Univerzitna nemocnica Bratislava, Nemocnica Ruzinov | Bratislava |  | 82606 | Slovakia |
| 4502 | EK-Fakultna nemocnica Trencin | Fakultna nemocnica Trencin | Trencin |  | 91101 | Slovakia |
| 4504 | EK-Ustredna vojenska nemocnica SNP Ruzomberok | Ustredna vojenska nemocnica SNP Ruzomberok- Fakultna nemocnica | Ruzomber ok |  | 034 26 | Slovakia |
| 4507 | EK-Fakultna nemocnica s poliklinikou F.D. Roosevelta | NsP Banská Bystrica | Banska Bystrica |  | 975 17 | Slovakia |
| 4503 | EK-Fakultna nemocnica Zilina | Fakultna nemocnica s poliklinikou Zilina | Zilina |  | 012 07 | Slovakia |
| 4501 | Eticka komisia NsP Trebisov a.s. | Nemocnica s poliklinikou Trebisov a.s. | Trebisov |  | 075 01 | Slovakia |
| 4510 | EK-UN Bratislava, Nemocnica sv. Cyrila a Metoda | Univerzitna nemocnica Bratislava, Nemocnica sv. Cyrila a Metoda | Bratislava |  | 85107 | Slovakia |
| 4512 | EK-Nemocnica Poprad a.s. | Nemocnica Poprad a.s. | Poprad |  | 058 45 | Slovakia |
| 4513 | EK-FNsP Nove Zamky | Fakultna nemocnica s poliklinikou Nove Zamky | Nove Zamky |  | 94002 | Slovakia |
| 4514 | Oftal s.r.o, Specializovana nemocnica v odbore oftalmologia | Oftal s.r.o. | Zvolen |  | 960 01 | Slovakia |
| 4517 | Rozhodnutie Etickej komisie Nemocnica Svateho Michala, a.s., | Nemocnica svateho Michala | Bratislava |  | 811 08 | Slovakia |
| 4900 | Ankara University Ethics Committee | Hacettepe University Medical Faculty | Ankara |  | 061 00 | Turkey |
| 4901 | Ankara University Ethics Committee | Ankara University Medical Faculty | Ankara |  | 061 00 | Turkey |
| 4902 | Ankara University Ethics Committee | Bilim University Florence Nightingale Hospital | Ankara |  | 061 00 | Turkey |
| 4904 | Ankara University Ethics Committee | Gazi University Hospital | Ankara |  | 061 00 | Turkey |
| 4903 | Ankara University Ethics Committee | Ankara Ataturk Training and Research Hospital | Ankara |  | 061 00 | Turkey |
| 8700 | State Institution "The Filatov Institute of Eye Diseases and Tissue Therapy of the National Academy of Medical Sciences of Ukraine" | V.P.Filatov Institute of Eye Diseases and Tissue Therapy AMS | Odessa |  | 65061 | Ukraine |
| 8701 | Local Ethics Committee at the Oppthalmology Clinic Eye Microsurgery Center | City clinical ophthalmological hospital | Kyiv |  | 3680 | Ukraine |
| 8702 | Local Ethics Committee at the Communal Institution Dnipropetrovsk Regional Clinical Ophthalmological Hospital | Regional clinical hospital n.a.  Mechnikova | Dnipropetr ovsk |  | 49005 | Ukraine |
| 8703 | Local Ethics Committee at the Hospital | LLC “Lugansk Regional Central Eye Hospital” | Lugansk |  | 91055 | Ukraine |

| **Center Number** | **Ethics Committee or Institutional Review Board** | **Department / Organization** | **EC/IRB**  **City** | **EC/IRB**  **State/ Province** | **EC/IRB**  **Postal Code** | **Center Country** |
| --- | --- | --- | --- | --- | --- | --- |
| 8704 | Local Ethics Committee at the Kharkov Regional Clinical Hospital | Kharkov Regional Clinical Hospital | Kharkiv |  | 61022 | Ukraine |
| 9000 | Comité de Etica Independiente de la Fundación Dominicana de Infectología | Consultorio Oftalmológico Medicalnet | Santo Domingo | Santo Domingo | N/A | Dominican Republic |
| 9001 | Comité de Etica del Centro Cardio-Neuro Oftalmológico y Trasplante CECANOT | Centro Cardio-Neuro Oftalmológico y Trasplante (CECANOT) | Santo Domingo | Santo Domingo | N/A | Dominican Republic |
| 9031 | Instituto Conmemorativo Gorgas de Estudios de la Salud | Clínica de Vitreo y Macula Dra. Ana Paz | Panamá | Panamá | N/A | Panamá |
| 9033 | Instituto Conmemorativo Gorgas de Estudios de la Salud | Clinica Yee | Panamá | Panamá | N/A | Panamá |
| 9051 | Comité de Etica Zugueme | Clínica Oftalmológica Santa Clara | Guatemala | Guatemal a | 1015 | Guatemala |
| 9061 | Research Ethics Committee - Central Directorate for Research and Health Development | Ministry of Health and Population | Cairo | Cairo | 11516 | Egypt |
| 9062 | Research Ethics Committee - Central Directorate for Research and Health Development | Ministry of Health and Population | Cairo | Cairo | 11516 | Egypt |
| 9064 | Research Ethics Committee - Central Directorate for Research and Health Development | Ministry of Health and Population | Cairo | Cairo | 11516 | Egypt |
| 9065 | Research Ethics Committee - Central Directorate for Research and Health Development | Ministry of Health and Population | Cairo | Alexandria | 11516 | Egypt |
| 9066 | Research Ethics Committee - Central Directorate for Research and Health Development | Ministry of Health and Population | Cairo | Cairo | 11516 | Egypt |
| 9071 | Instituto Costarricense de Investigaciones Clínicas (ICIC) | Instituto de Cirugia Ocular | San José | San José | N/A | Costa Rica |
| 9072 | Instituto Costarricense de Investigaciones Clínicas (ICIC) | Clínica 20/20 | San José | San José | N/A | Costa Rica |
| 9073 | Instituto Costarricense de Investigaciones Clínicas (ICIC) | Oftalmocima | San José | San José | N/A | Costa Rica |
| 1500 | Centro Nacional de Bioetica | Unidad Oftalmologica de Caracas, C.A. Santa Paula | Caracas |  | 1060 | Venezuela |
| 1501 | Centro Nacional de Bioetica | Clinica de Especialidades Oftalmologicas (Retina & Vitreo) | Caracas |  | 1060 | Venezuela |
| 1502 | Centro Nacional de Bioetica | Centro Oftalmologico de Valencia (CEOVAL) | Caracas |  | 1060 | Venezuela |
| 1503 | Centro Nacional de Bioetica | Instituto Oftalmológico IUMO trinidad | Caracas |  | 1060 | Venezuela |
