# Supplementary material for: Real-world effectiveness and safety of ranibizumab for the treatment of myopic choroidal neovascularization: Results from the LUMINOUS study
Source: PLoS One. 2020 Jan 21;15(1):e0227557. doi: 10.1371/journal.pone.0227557 (PMC6974143; doi:10.1371/journal.pone.0227557)
Supplement: S2 Table — (DOCX) [file pone.0227557.s004.docx]

**S2 Table. Ocular adverse events suspected to be related to ranibizumab in treatment-naïve and prior ranibizumab-treated patients with mCNV at 1 year.**

| **Preferred term, n (%)** | **Treatment-naïve, n=108** | **Prior ranibizumab-treated, n=175** |
| --- | --- | --- |
| **Ocular AEs, total** | **3 (2.78)** | **6 (3.43)** |
| Conjunctival hemorrhage | 0 | 2 (1.14) |
| Metamorphopsia | 1 (0.93) | 0 |
| Retinal pigment epithelial tear | 1 (0.93) | 0 |
| Vitreous floaters | 0 | 1 (0.57) |
| Corneal abrasion | 0 | 1 (0.57) |
| Post procedural inflammation | 0 | 1 (0.57) |
| Increased IOP | 1 (0.93) | 1 (0.57) |

Indication and pre-treatment status refers to the primary treated eye. Only AEs occurring during the safety observation period are included. Preferred terms are presented by descending order of frequency in the total column. A patient with multiple occurrences of an AE was counted once per preferred term. A patient with multiple AEs is counted only once in the total row. Patients with a baseline visit date present are included. Data collected until the last recorded follow-up date was used to perform the analyses.

AE, adverse events; IOP, intraocular pressure; mCNV, myopic choroidal neovascularization; n, number of patients
